# Supplementary material for: The centromere landscapes of four karyotypically diverse Papaver species provide insights into chromosome evolution and speciation
Source: Cell Genom. 2024 Jul 30;4(8):100626. doi: 10.1016/j.xgen.2024.100626 (PMC11406182; doi:10.1016/j.xgen.2024.100626)
Supplement: Document S1. Figures S1–S30 and Tables S3, S7, and S10 [file mmc1.pdf]

Cell Genomics, Volume *4*

Supplemental information

The centromere landscapes of four karyotypically diverse *Papaver* species provide insights into chromosome evolution and speciation

Shenghan Gao (高胜寒), Yanyan Jia (贾彦彦), Hongtao Guo (郭弘涛), Tun Xu (徐暾), Bo Wang (王博), Stephen J. Bush, Shijie Wan (万世杰), Yimeng Zhang (张一蒙), Xiaofei Yang (杨晓飞), and Kai Ye (叶凯)

Supplemental information

The centromere landscapes of four karyotypically diverse *Papaver* species provide insights into chromosome evolution and speciation

Shenghan Gao, Yanyan Jia, Hongtao Guo, Tun Xu, Bo Wang, Stephen J. Bush, Shijie Wan, Yimeng Zhang, Xiaofei Yang, Kai Ye

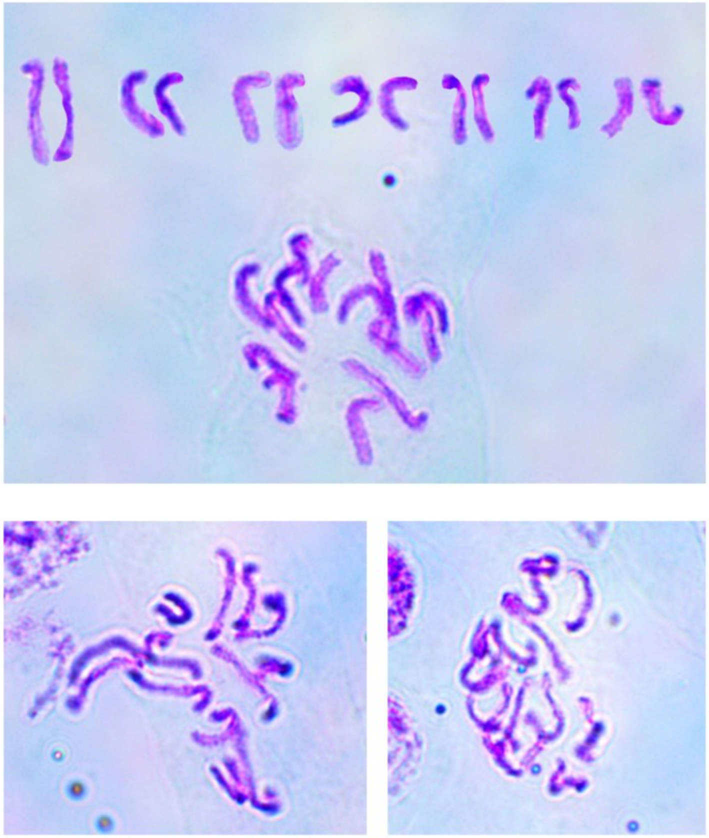

Figure S1. Karyotyping of *P. bracteatum*, related to STAR Methods. We independently repeated the karyotyping experiment three times and obtained identical results.

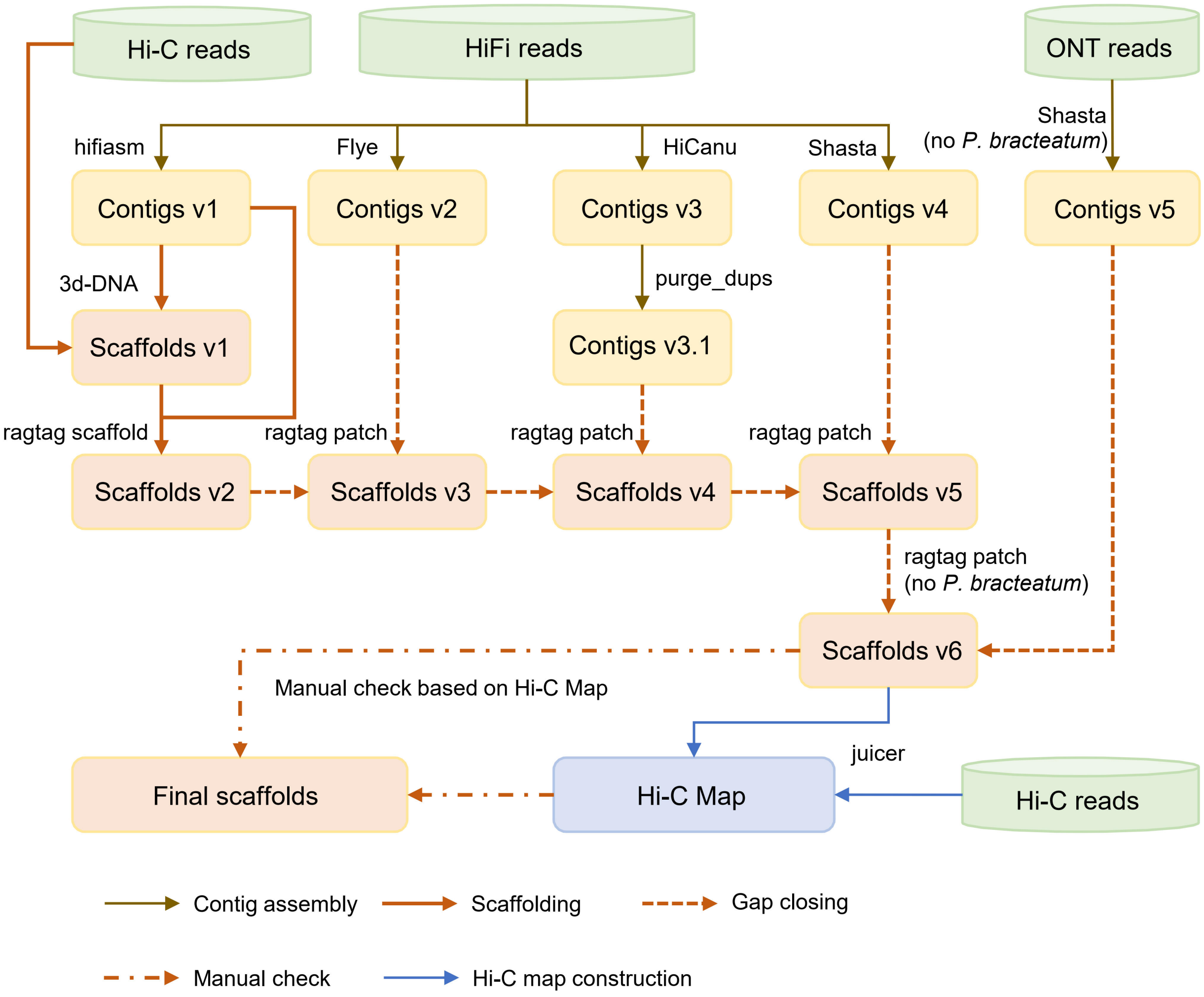

Figure S2. Genome assembly pipeline, related to Table 1. First, we generated contigs by four independent assembly tools, including hifiasm (v0.16.1), Flye (v2.7-b1585), HiCanu (v2.1.1), and Shasta (v0.10). We generated initial scaffolds (scaffolds v1) via 3d-DNA (v180419) based on hifiasm contigs and Hi-C reads. And then the ragtag (v2.0.1) scaffold was applied to refine the scaffolds v1 to scaffold v2. Next, we iteratively closed gaps via ragtag path command to generate scaffold v6. Then, we aligned Hi-C reads to scaffolds v6 and constructed the Hi-C map by juicer (v1.5.7). And finally, we manually corrected mis-assemblies based on Hi-C contact map.

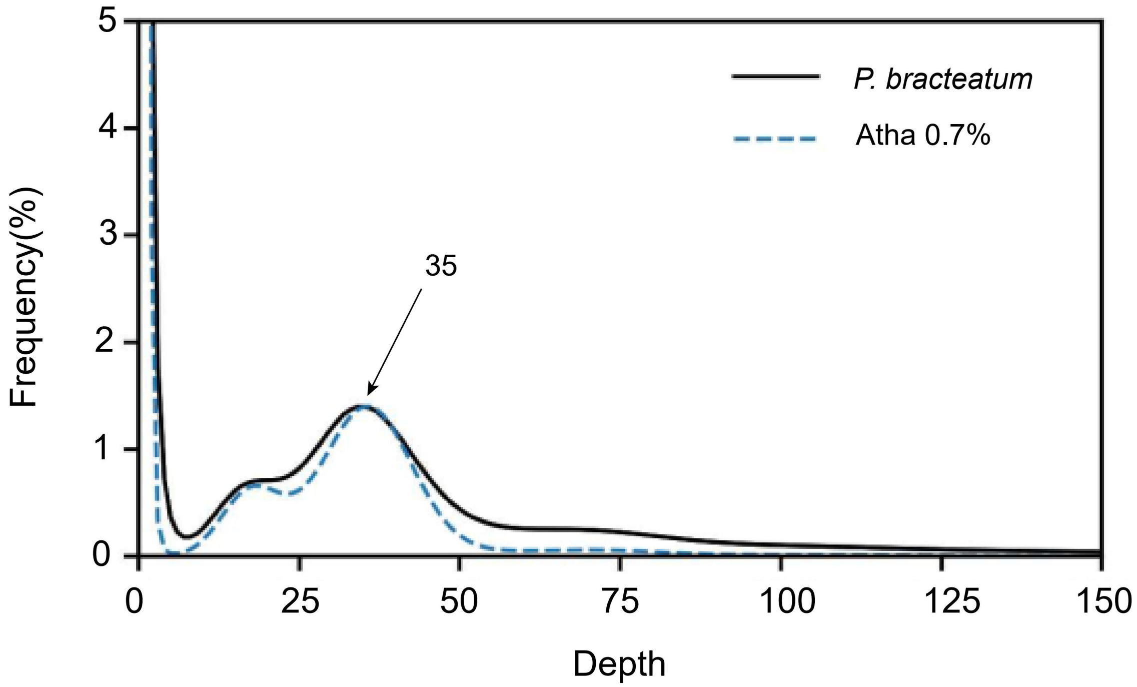

Figure S3. Genome size estimation of *P. bracteatum*, related to Table 1. K-mer frequency distributions from Illumina short-reads. With K=17, there is a major peak value at 35 of *P. bracteatum*. The dash blue line is K-mer frequency distribution of simulated 31X Illumina paired-end sequencing of *Arabidopsis thaliana* with 0.7% heterozygosity.

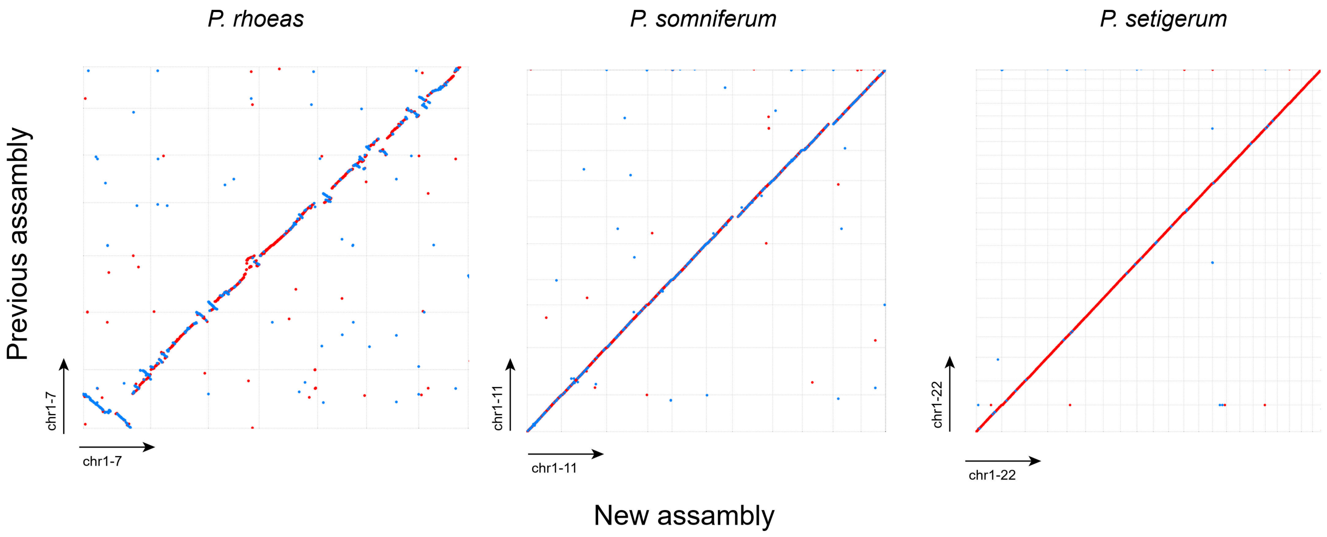

Figure S4. Genome comparison between assemblies in this work and previous published assemblies, related to Table 1. The dotplots generated by Mummer (v4.0.0)**.**

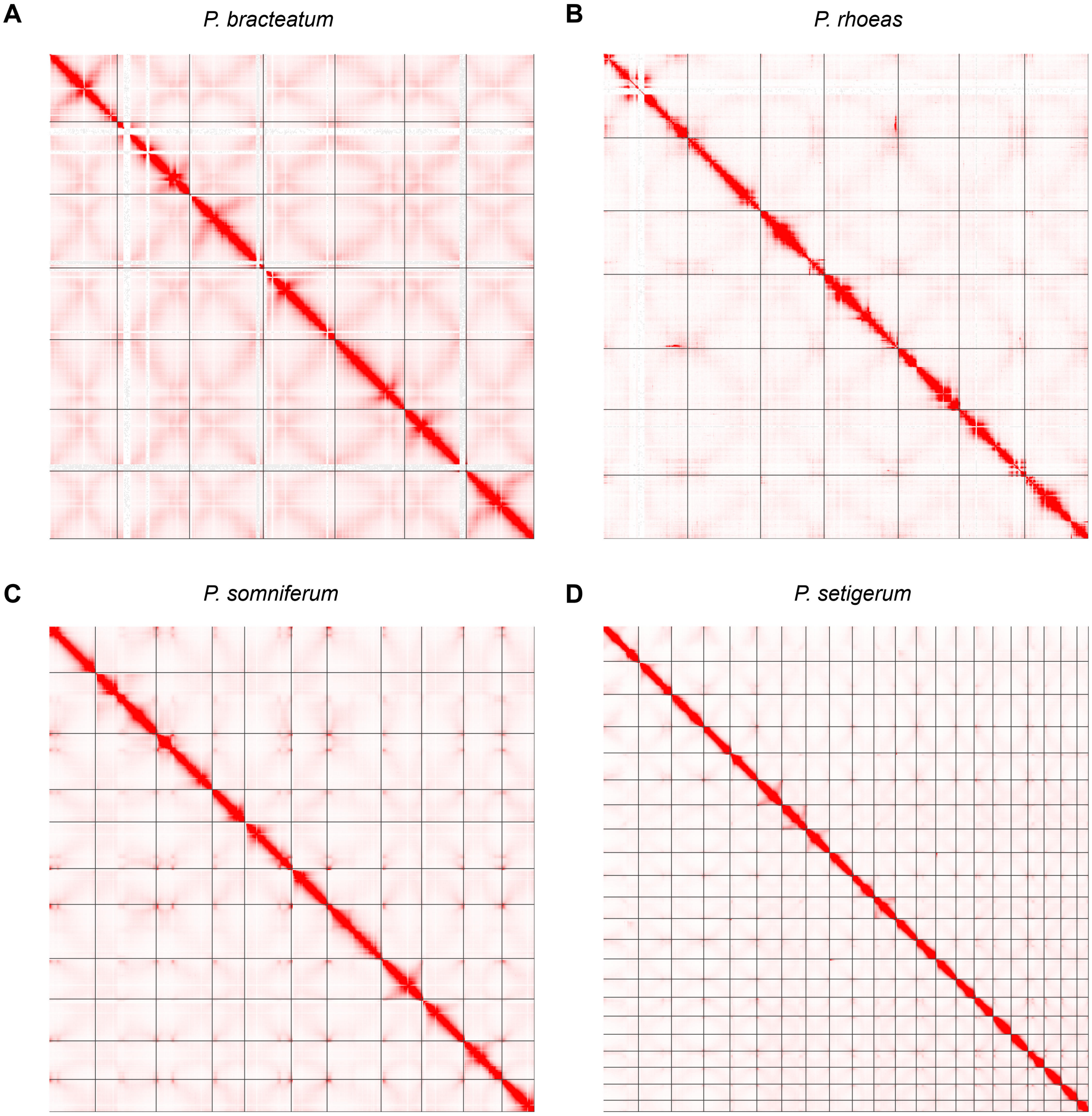

Figure S5. Hi-C contact heatmap of four genomes, related to Table 1. The results indicated the high-quality genome assemblies of *P. bracteatum* (**A**), *P. rhoeas* (**B**), *P. somniferum* (**C**), and *P. setigerum* (**D**). The contact maps are generated by juicer (v1.5.7).

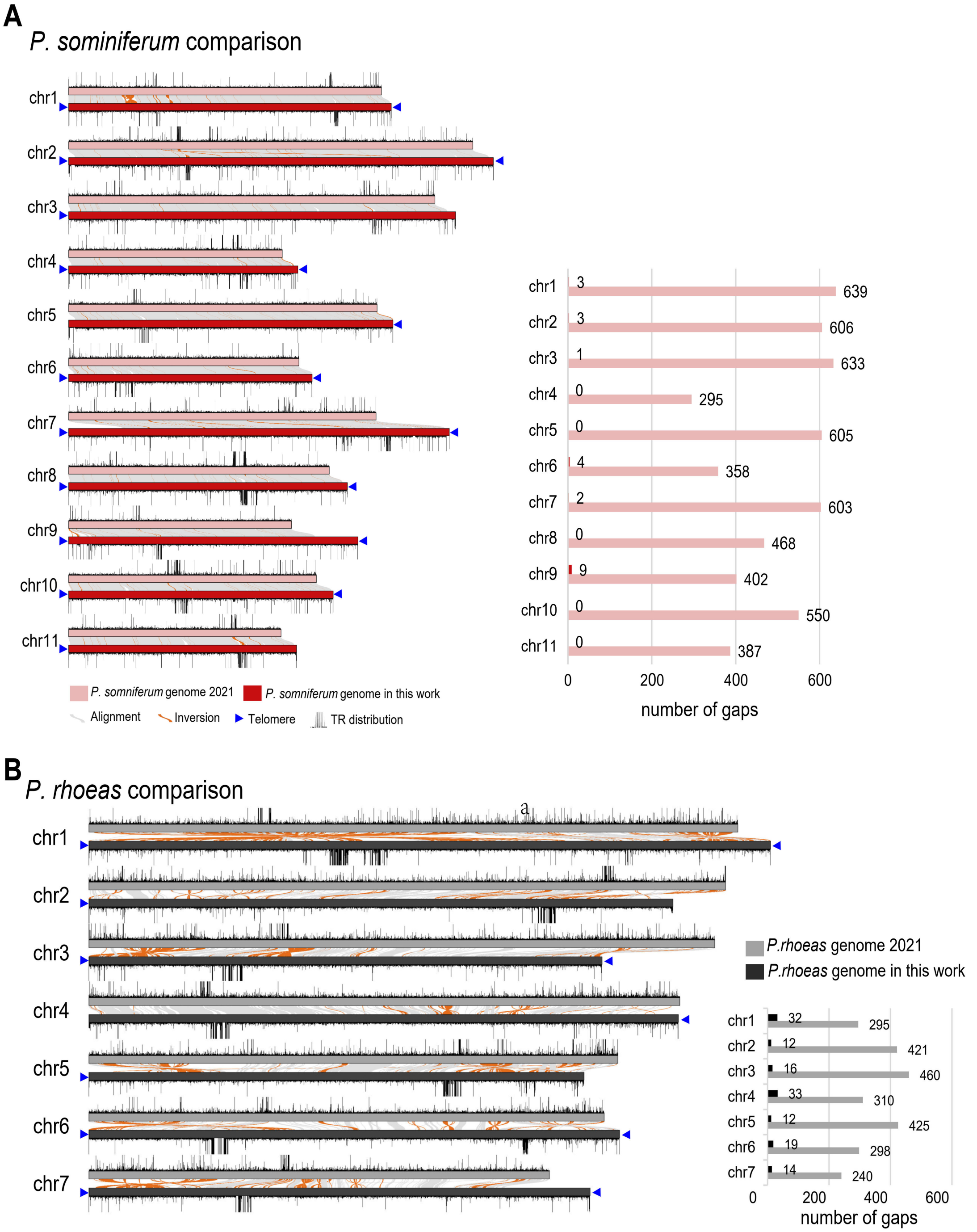

Figure S6. Comparison between genomes in this work and that published in 2021, related to Figure 1. (**A**) Comparison for *P. somniferum*. (**B**) Comparison for *P. rhoeas*. We showed the assembled telomeres as blue triangles, the tandem repeat (TR) density for each chromosome is visualized and the number of gaps showed in the barplots.

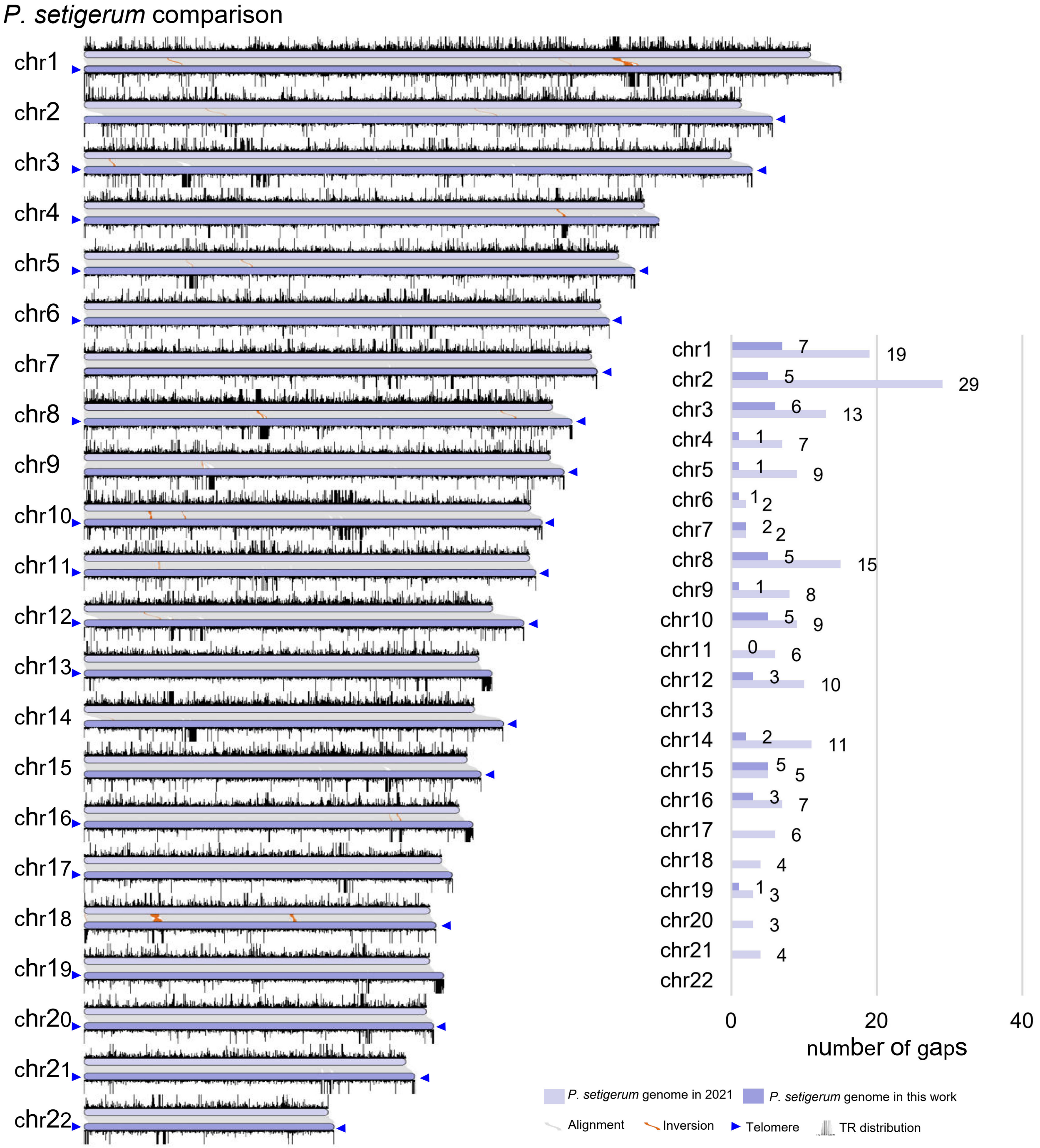

Figure S7. Comparison between *P. setigerum* genomes in this work and that published in 2021, related to Figure 1. We showed the assembled telomeres as blue triangles, the tandem repeat (TR) density for each chromosome is visualized and the number of gaps showed in the barplots.

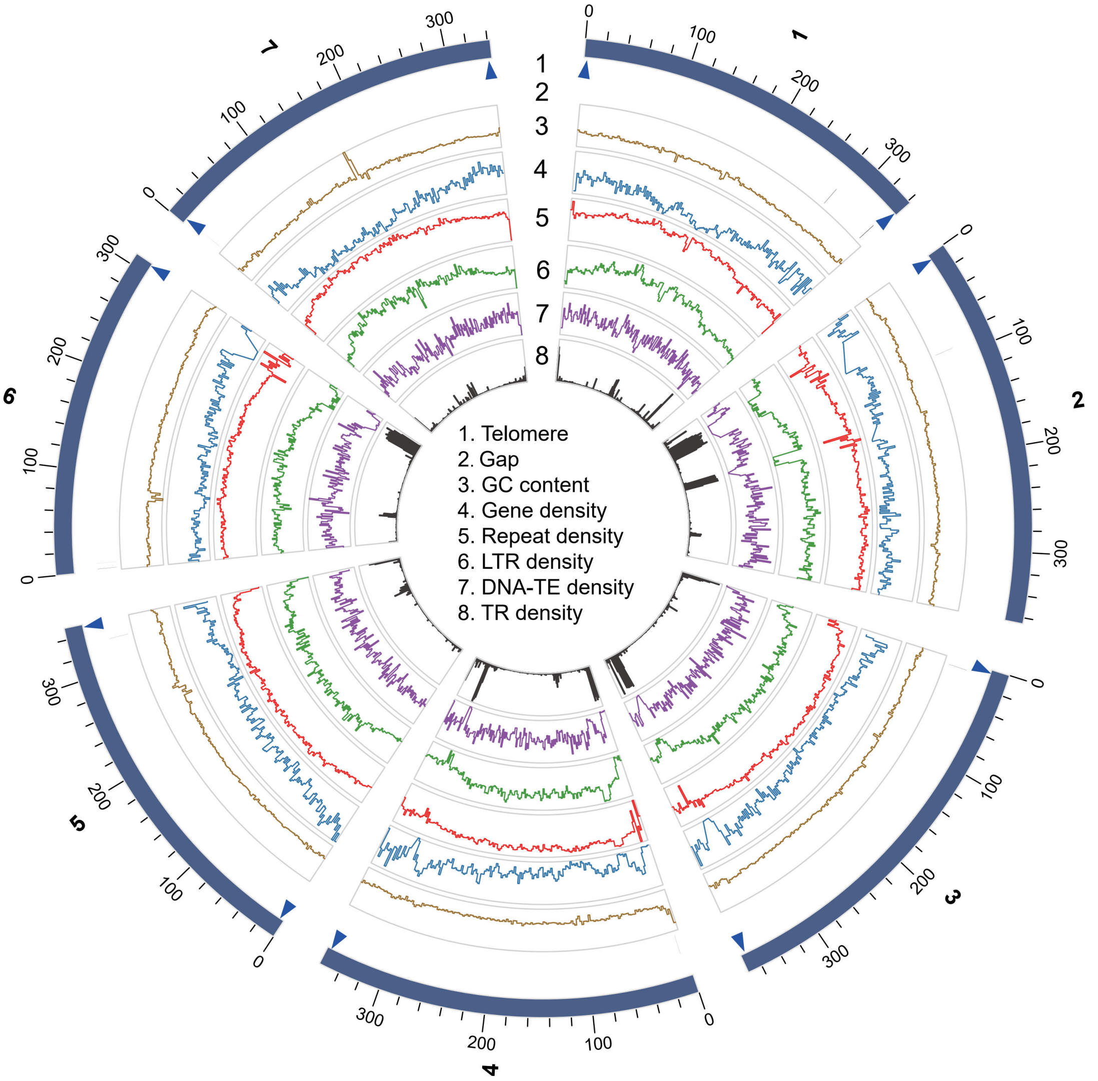

Figure S8. Genome assembly and genetic features of *P. bracteatum*, related to Figure 1. Circos plot of *P. bracteatum* assembly shows genomic features. Tracks one to eight represent the telomere sign (11 telomeres were assembled in total), assembly gaps (18 in total), GC content, Gene density, Repeat density, LTR density, DNA-TE density, and tandem repeat (TR) density, respectively.

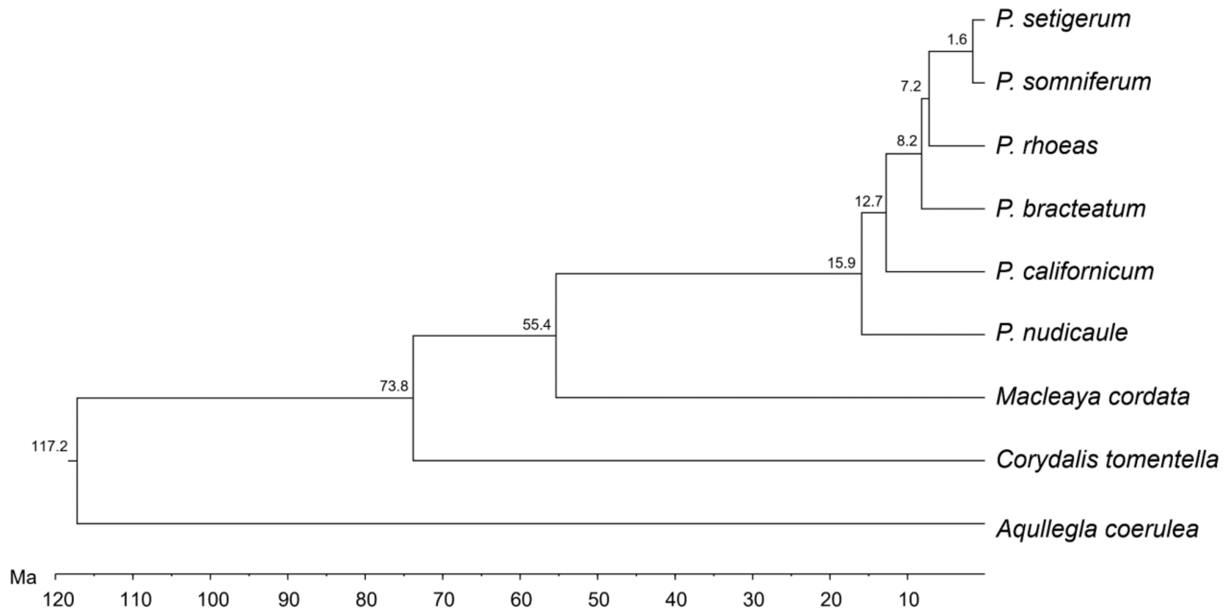

Figure S9. Phylogenetic tree of nine species, related to STAR Methods. Inferred phylogenetic tree with 13 single-copy orthologs of 9 species identified by OrthoFinder (v2.3.4). Single-copy ortholog pairs were aligned with MAFFT (v7), with the conserved sites in the alignments extracted using Gblocks (v0.91b) with the default parameters, followed by maximum likelihood phylogenomic tree construction using RAxML (v8.2.12) with 100 bootstraps. The divergence times between species were estimated using r8s (v.1.8) with the penalized likelihood method and parameter 'setsmoothing = 1000'.

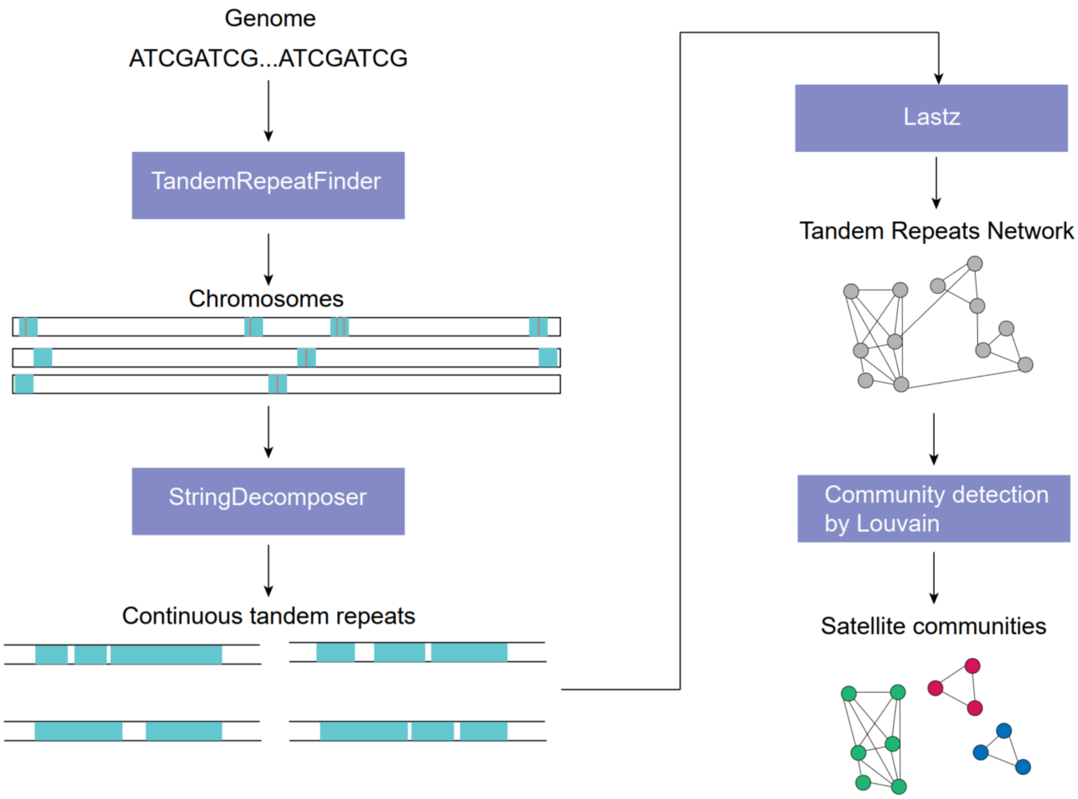

Figure S10. Satellite library construction pipeline, related to Figure 2. We first applied tandem repeat finder (TRF) (v4.09) to detected tandem repeats (TRs) (light blue block). Then, StringDecomposser was applied on each TR region to obtain tandem repeat units. We construct a TR similarity network based on Lastz alignment and detected the communities by Louvain methods. Each community represented a satellite.

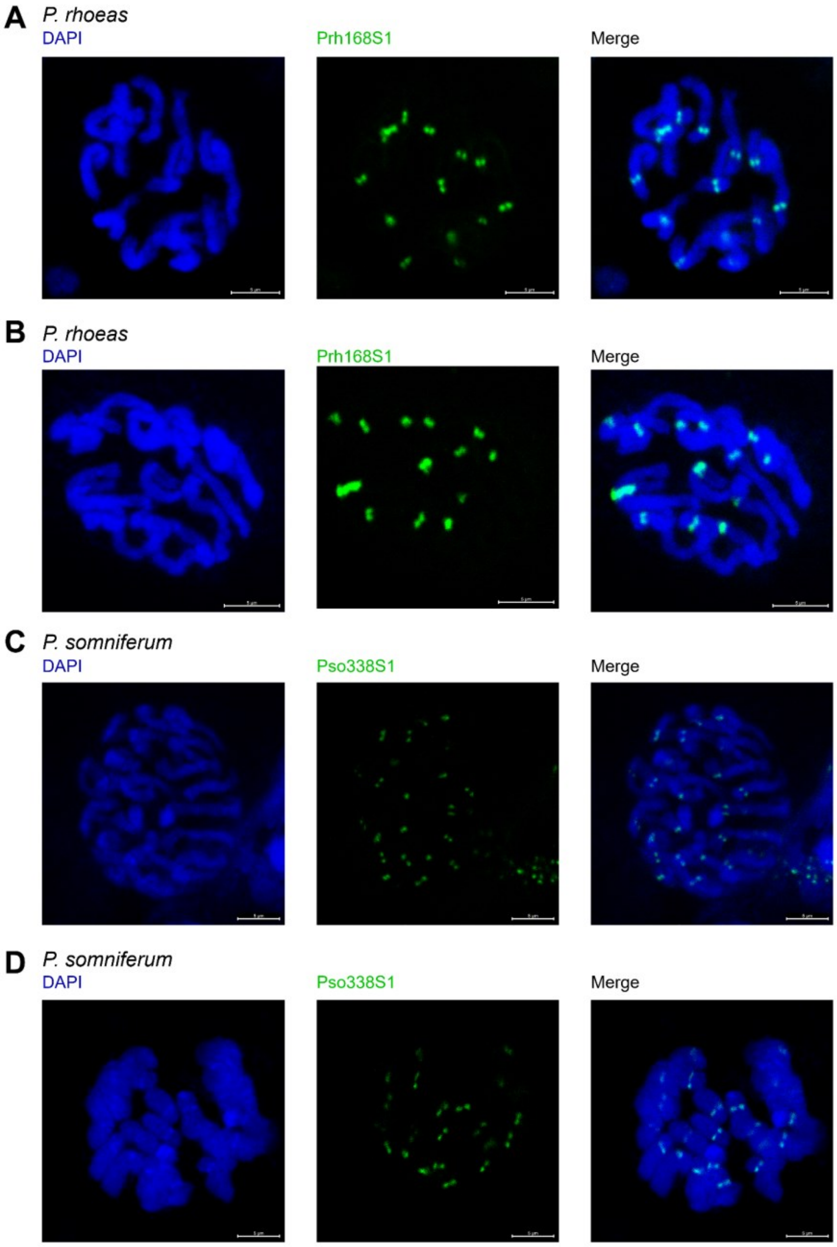

Figure S11. Fluorescence in situ hybridization (FISH) of Prh168S1 and Pso338S1 on metaphase chromosomes in *P. rhoeas* and *P. somniferum*, respectively, related to Figure 2. Metaphase chromosomes stained with 4′,6-diamidino-2-phenylindole (DAPI) (blue, Left), Prh168S1 and Pso338S1 FISH probes (green, middle). We independently repeated the FISH experiment three times and obtained similar results.

A

```
CLUSTAL O(1.2.4) multiple sequence alignment


Pso04G02820.1              MARKKHFAQRYTPGGRQ--------PP---PP------------------TPPPPS---- 27
Prh03G45160.1              MARRKHFAQRYPPGGRQ--------PQ---PP------------------PPPPPP---- 27
NnCenH3-A                  MARTKHLFRKKTTQSSRLSSAAGVSPASPATP----------------------APVENA 38
sp|Q8RVQ9|HTR12_ARATH      MARTKHRVTRSQPRN--QTDAAGASSSQAAGPTTTPTRRGGEGGDNTQQTNPTTSPATGT 58
                           *** **   :    .                *

Pso04G02820.1              -----AAGSSSDAGGKKRSYRHKPGAKALQEIRKLQKNIDLLLPRAPFVRIVKEITDNF- 81
Prh03G45160.1              ------PSSSSDAAAKKRPYKRKPGTKALQDIRKLQKSIDLLMPRAPFVRIVKEITDNF- 80
NnCenH3-A                  RNKKADAPESSTKRQQHKPHRYRPGTVALREIRRYQKTWTLLIPAAPFIRTVKEISNFY- 97
sp|Q8RVQ9|HTR12_ARATH      RRGAKRSRQAMPRGSQKKSYRYRPGTVALKEIRHFQKQTNLLIPAASFIREVRSITHMLA 118
                                   .:     ::: :: :**: **::**: **   **:* * *:* *:.*:.

Pso04G02820.1              SKEVNRWQAEALTALQEATEAFLVNTFEDAQLCAIHAKRVTIMQKDWQLARRLGGRGHYG 141
Prh03G45160.1              SKEVNRWQAEALTALQEAAEAFLVGTFQDAQLCAIHAKRVTIMQKDWQLARRLGGRGQYG 140
NnCenH3-A                  SPEVTRWTAEALVALQEAAEDYLVHLFEDAMLCAIHAKRVTLMQKDWALARRLGGKGQLR 157
sp|Q8RVQ9|HTR12_ARATH      PPQINRWTAEALVALQEAAEDYLVGLFSDSMLCAIHARRVTLMRKDFELARRLGGKGRPW 178
                             ::.** ****.*****:* :**  *.*: ******:***:*:**: *******:*:

Pso04G02820.1              SQPW 145
Prh03G45160.1              SQPW 144
NnCenH3-A                  ---- 157
sp|Q8RVQ9|HTR12_ARATH      ---- 178
```

CENH3

B

	Pso04G02820.1	NnCenH3-A	HTR12_ARATH
**Prh03G45160.1**	83.45% (top 1 hit)	66% (top 1 hit)	54.95% (top 1 hit)

Figure S12. Identification of *PrhCENH3* gene as *Prh03G45160.1*, related to Figure 2. (**A**) Cluster Omega alignment of protein sequences of *Prh03G45160.1, Pso04G02820.1 (PsoCENH3, CENH3* in *P. somniferum), NnCenH3-A, and HTR12_ARATH.* EALT is the sequence feature of CENH3 in *P. somniferum* and *P. rhoeas*. (**B**) The BlastP alignments between the protein sequences of Prh03G45160.1 and other three CENH3, Pso04G02820.1, NnCenH3-A, HTR12_ARATH. The percents indicate the identities.

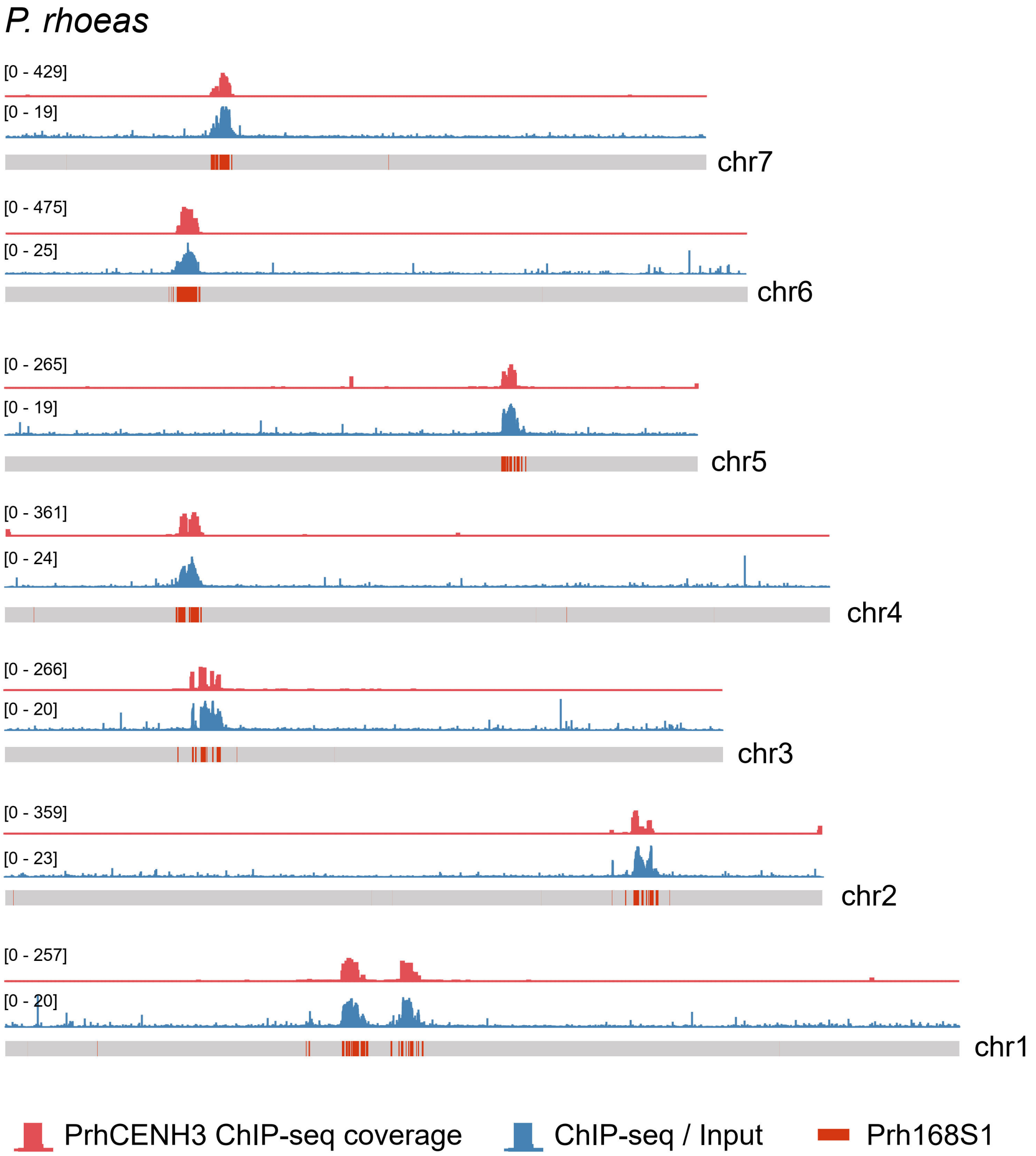

Figure S13. The validation of Prh168S1 centromere satellite by PrhCENH3 ChIP-seq, related to Figure 2. The Prh168S1, PrhCENH3 ChIP-seq coverage, and the ChIP-seq/input ratio are shown on each chromosome of *P. rhoeas*.

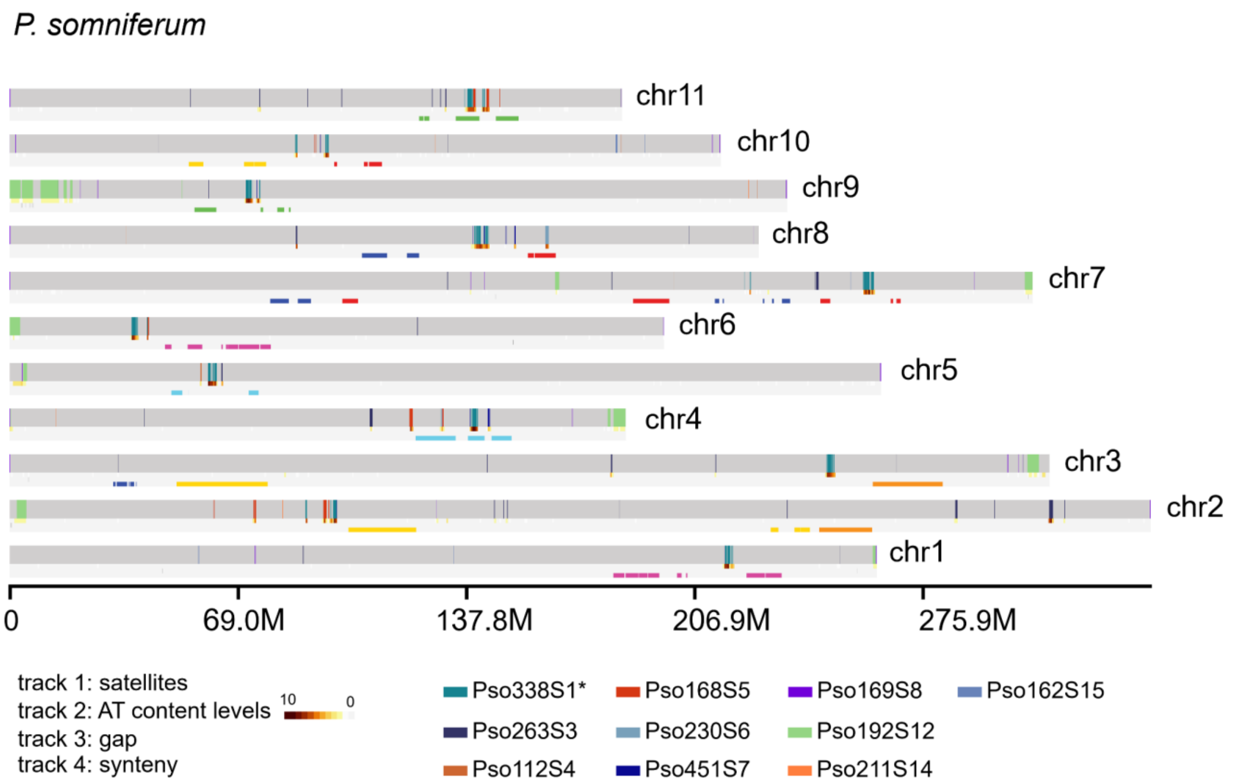

Figure S14. The genomic landscape of satellites in *P. somniferum*, related to Figure 2. The satellites with AT content levels, gaps and syntenic relations associated with pericentromeric regions in *P. rhoeas* are visualized. The pericentromeric syntenic relations are highlighted by different colors. Satellites with "*" represent predicted centromere satellites.

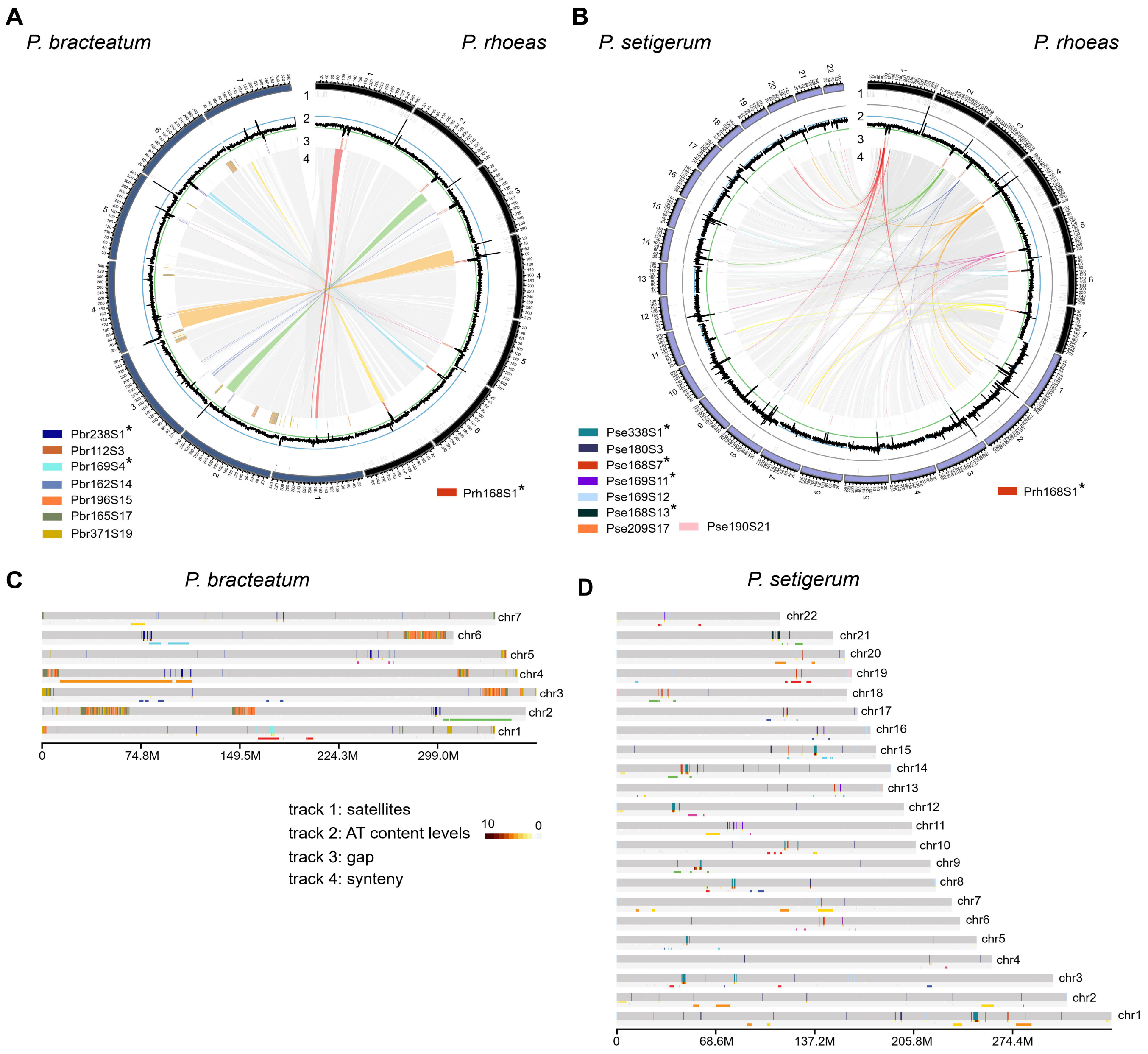

Figure S15. Prediction of centromeres in *P. bracteatum* and *P. setigerum*, related to Figure 2. Centromere satellite prediction in *P. bracteatum* (**A**) and *P. setigerum* (**B**) based on filtered satellite library and pericentromeric syntenic relations with *P. rhoeas*. The tracks 1, 2, 3, and 4 indicate assembly gaps, tandem repeat density, satellites, and syntenic relations, respectively. The syntenic relations are detected by DRIMM-Synteny. The pericentromeric syntenic relations are highlighted by different colors. Satellites with "*" represent predicted centromere satellites. The genomic landscapes of satellites with AT content levels, gaps and syntenic relations associated with pericentromeric regions of *P. rhoeas* in *P. bracteatum* (**C**) and *P. setigerum* (**D**).

A

```
CLUSTAL O(1.2.4) multiple sequence alignment


Pso03G15860.1           MARTKQTARKSTG----------------------------GKAPRK----------QLA 22
Pso03G15540.1           MARTKQTARKSTG----------------------------GKAPRK----------QLA 22
Pso11G43230.1           MARTKQTARKSAG----------------------------GKALRK----------QLA 22
Pso09G04460.1           MARTKQTARKSTG----------------------------GKAPRK----------QLA 22
Pso03G52660.1           MARTKQTARKSTG----------------------------GKAPRK----------QLA 22
Pso02G11060.1           MARTKQTARKSTG----------------------------GKAPRK----------QLA 22
sp|Q8RVQ9|HTR12_ARATH   MARTKHRVTRSQPRNQTDAAGASSSQAAGPTTTPTRRGGEGGDNTQQTNPTTSPATGTRR 60
NnCenH3-A               MARTKHLFRKKTTQSSRLSSAAGVSP--------------------ASPATPAPVENARN 40
Pso04G25820.1           MSRKKHFARRSGG--------VPGQQ--------------------P-PSS--------- 22
Pso05G19150.1           MSRKKHFARRSGG--------VPGQQ--------------------P-PSS--------- 22
Pso04G02820.1           MARKKHFAQRYTP--------GGRQP--------------------P-PPTPPPPS---- 27
Pso08G02370.1           MARKKHFAQRYTP--------GGRQQ--------------------L-PPPP-------- 23
                        *:*.*:   :

Pso03G15860.1           TKAARKSAPATGGVKKPHRFRPGTVALREIRKYQKSTELLIRKLPFQRLVREIAQDFK-- 80
Pso03G15540.1           TKAARKSAPATGGVKKPHRFRPGTVALREIRKYQKSTELLIRKLPFQRLVREIAQDFK-- 80
Pso11G43230.1           TKAARKSAPTTGGVKKPHRYRPGTVALREIRKYQKSTELLIRKLPFQRLVREIAQDFK-- 80
Pso09G04460.1           TKAARKSAPTTGGVKKPHRYRPGTVALREIRKYQKSTELLIRKLPFQRLVREIAQDFK-- 80
Pso03G52660.1           TKAARKSAPTTGGVKKPHRYRPGTVALREIRKYQKSTELLIRKLPFQRLVREIAQDFK-- 80
Pso02G11060.1           TKAARKSAPTTGGVKKPHRYRPGTVALREIRKYQKSTELLIRKLPFQRLVREIAQDFK-- 80
sp|Q8RVQ9|HTR12_ARATH   GAKRSRQAMPRGSQKKSYRYRPGTVALKEIRHFQKQTNLLIPAASFIREVRSITHMLAPP 120
NnCenH3-A               KKADAPESSTKRQQHKPHRYRPGTVALREIRRYQKTWTLLIPAAPFIRTVKEISNFYS-P 99
Pso04G25820.1           ------SSQPQAAQRKPYRHKPGTVALKEIRKFQKSFDLLLPRAPFIRIVKEITNNFS-K 75
Pso05G19150.1           ------SSQPQAAQRKPYRHKPGTVALKEIRKCQKSFDLLLPRAPFIRIVKEITNNFS-K 75
Pso04G02820.1           ---AAGSSSDAGGKKRSYRHKPGAKALQEIRKLQKNIDLLLPRAPFVRIVKEITDNFS-K 83
Pso08G02370.1           ----ASSSSDAGGKKRSYRHKPGTKALQEIRKLQKSIDLLLPRAPFVRIVKEITDNFS-K 78
                              .:      :: :*.:**: **:***: **   **:    * * *:.*:.

Pso03G15860.1           TDLRFQSSAVAALQEAAEAYLVGLFEDTNLCAIHAKRVTIMPKDIQLARRIRGERA---- 136
Pso03G15540.1           TDLRFQSSAVAALQEAAEAYLVGLFEDTNLCAIHAKRVTIMPKDIQLARRIRGERA---- 136
Pso11G43230.1           TDLRFQSHAVLALQEAAEAYLVGLFEDTNLCAIHAKRVTIMPKDIQLARRIRGERA---- 136
Pso09G04460.1           TDLRFQSHAVLALQEAAEAYLVGLFEDTNLCAIHAKRVTIMPKDIQLARRIRGERA---- 136
Pso03G52660.1           TDLRFQSHAVLALQEAAEAYLVGLFEDTNLCAIHAKRVTIMPKDIQLARRIRGERA---- 136
Pso02G11060.1           TDLRFQSHAVLALQEAAEAYLVGLFEDTNLCAIHAKRVTIMPKDIQLARRIRGERA---- 136
sp|Q8RVQ9|HTR12_ARATH   QINRWTAEALVALQEAAEDYLVGLFSDSMLCAIHARRVTLMRKDFELARRLGGKGR---- 176
NnCenH3-A               EVTRWTAEALVALQEAAEDYLVHLFEDAMLCAIHAKRVTLMQKDWALARRLGGKGQLR-- 157
Pso04G25820.1           DVDRWQAEALTALQEAAEAFLIHQFEDTQLCAIHAKRVTISNAKRLAA---------SKK 126
Pso05G19150.1           DVDRWQAEALTALQEEAEAFLIHLFEDTQLCAIHAKRVTISNAKRLAA---------SKK 126
Pso04G02820.1           EVNRWQAEALTALQEATEAFLVNTFEDAQLCAIHAKRVTIMQKDWQLARRLGGRGHYGSQ 143
Pso08G02370.1           EVNRWQAEALTALQEATEAFLVGTFEDAQLCAIHAKRVTIMQKDWQLARRLGGRGHYGSQ 138
                           *: : *: **** :* :*:  *.*: ******:***:   .   *

Pso03G15860.1           -------------      136
Pso03G15540.1           -------------      136
Pso11G43230.1           -------------      136
Pso09G04460.1           -------------      136
Pso03G52660.1           -------------      136
Pso02G11060.1           -------------      136
sp|Q8RVQ9|HTR12_ARATH   PW-----------      178
NnCenH3-A               -------------      157
Pso04G25820.1           AWWERSAMVRELH      139
Pso05G19150.1           AWWERSAMVREPQ      139
Pso04G02820.1           PW-----------      145
Pso08G02370.1           PW-----------      140
```

H3

H3.3

CENH3

B

	S1	S2	S3	S4	S5	S6	S7
Pso04G25820.1	0.96	0.63	0.77	1.93	1.20	1.35	1.03
Pso05G19150.1	0.80	0.34	0.84	0.97	0.61	0.66	0.71
Pso08G02370.1	1.62	1.18	2.36	2.86	1.44	2.21	3.06
Pso04G02820.1	7.01	4.18	9.22	10.51	8.54	9.91	11.04

Figure S16. CENH3 in *P. somniferum* (PsoCENH3) identification, related to Figure 2. (**A**) Multiple sequence alignment to identify candidate CENH3. SAVA is the sequence feature of H3 and HAVL is the sequence feature of H3.3. EALT is the sequence feature of CENH3 for *P. somniferum.* (**B**) The gene expression of four candidate CENH3s based on seven different seedling stages (S1 to S7). From these results, we identified *Pso04G02820.1* as the gene code PsoCENH3.

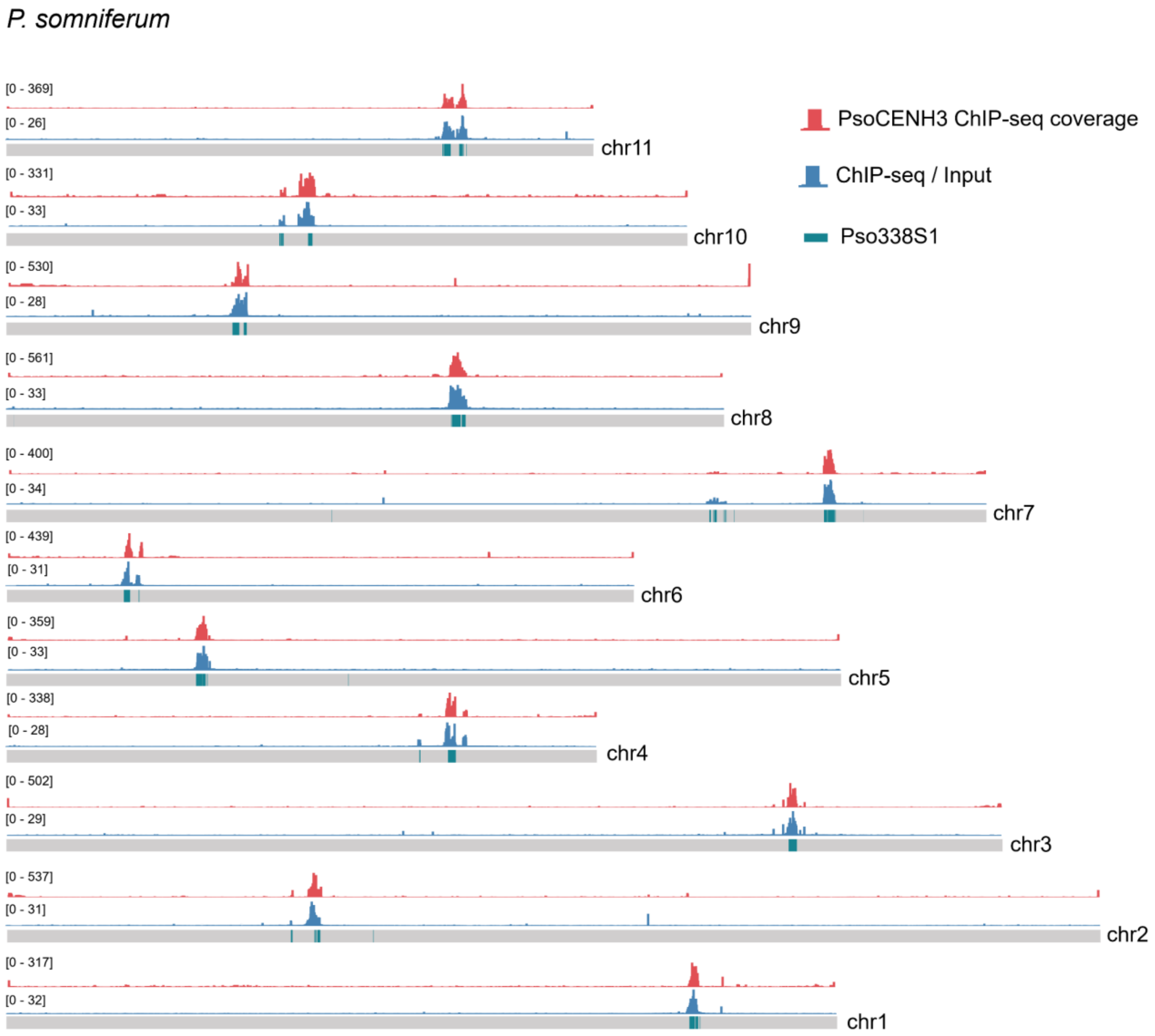

Figure S17. The validation of Pso338S1 centromere satellite, related to Figure 2. The Pso338S1, PsoCENH3 ChIP-seq coverage, and the ChIP-seq/input ratio are shown on each chromosome of *P. somniferum*.

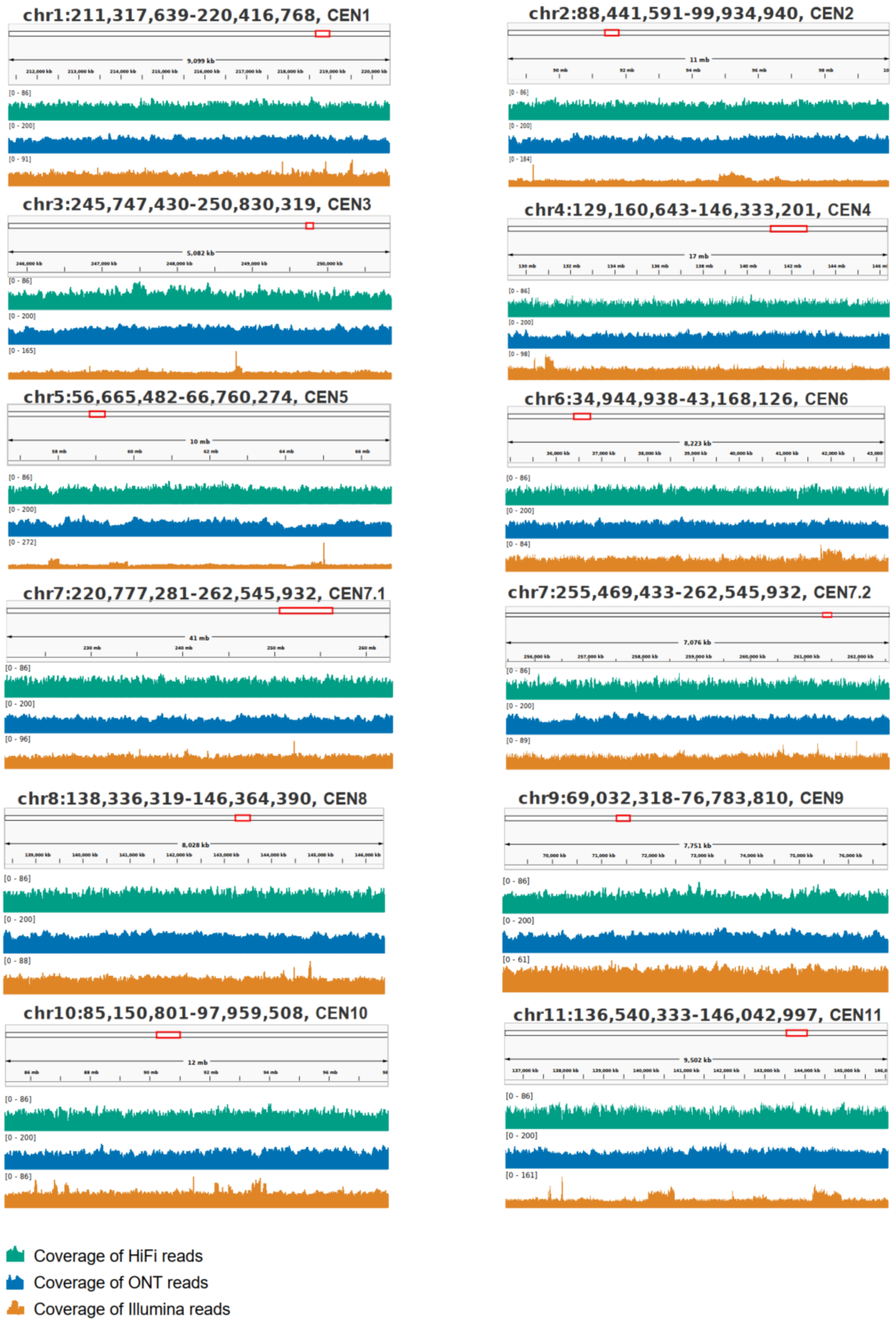

Figure S18. Sequencing coverage of HiFi, ONT, and Illumina reads in each centromere of *P. somniferum*, related to Figure 2.

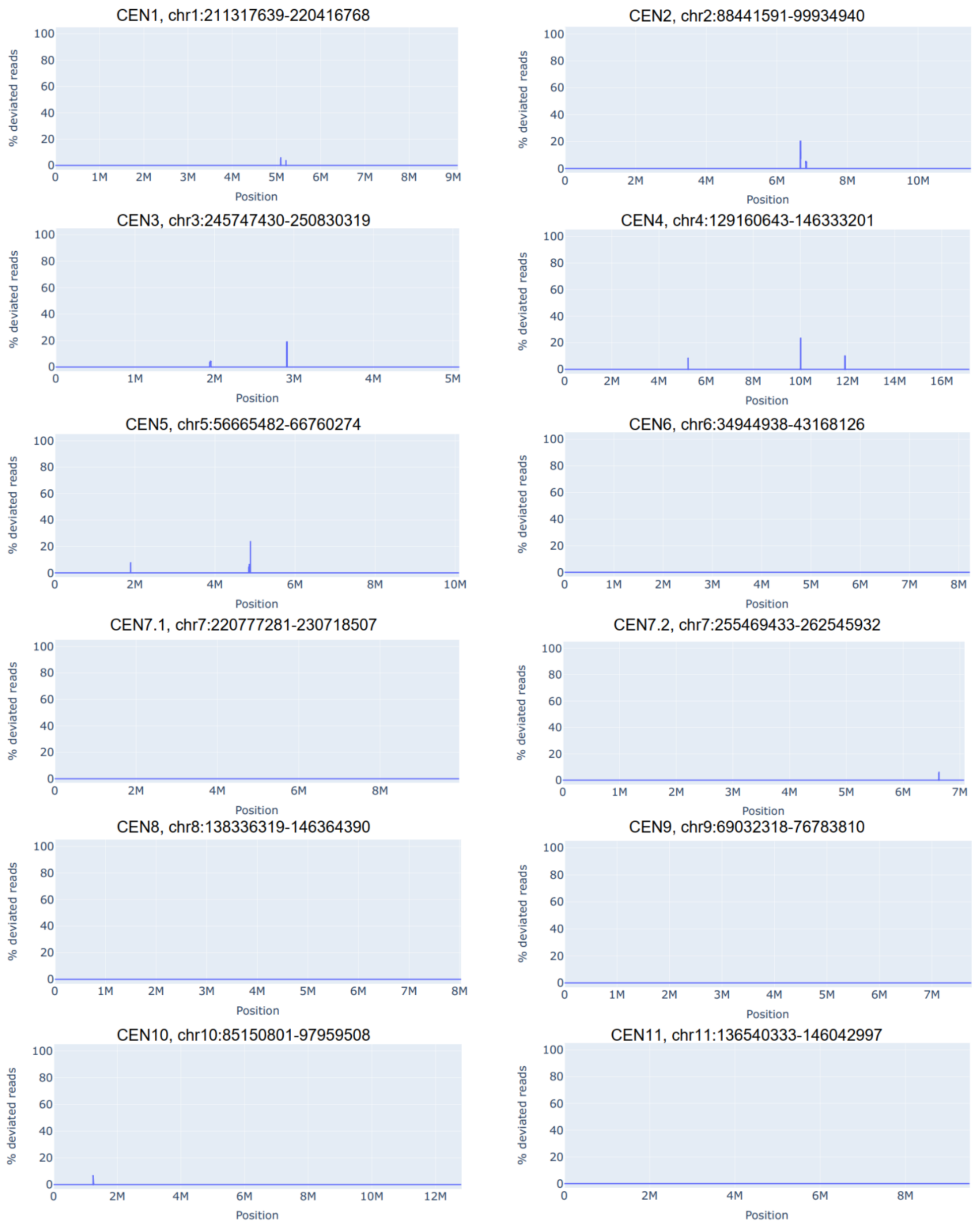

Figure S19. Assembly validation in each centromere of *P. somniferum* using VerityMap (v2.0.0), related to Figure 2.

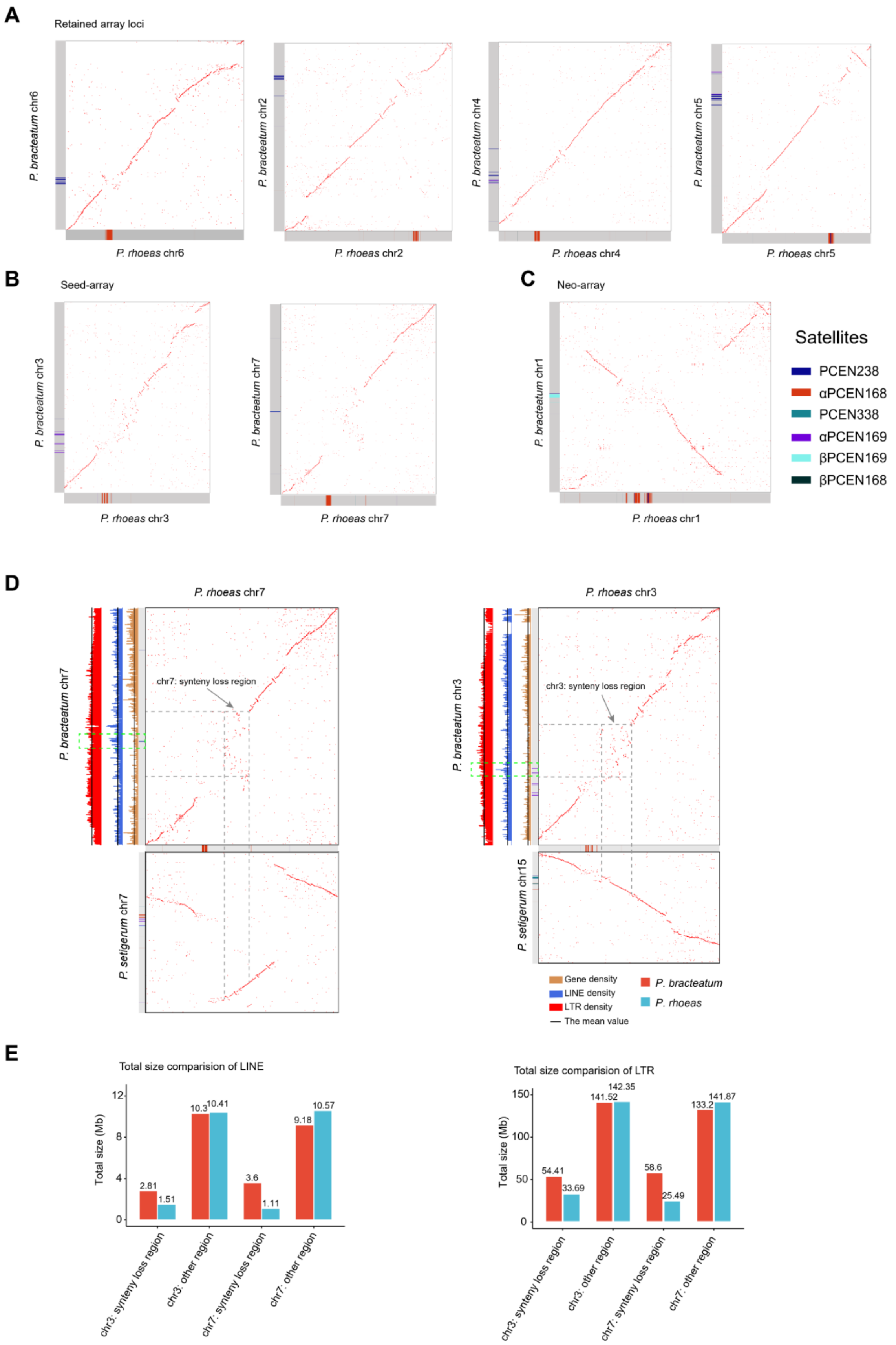
A
Retained array loci
P. bracteatum chr6
P. rhoeas chr6
P. bracteatum chr2
P. rhoeas chr2
P. bracteatum chr4
P. rhoeas chr4
P. bracteatum chr5
P. rhoeas chr5
B
Seed-array
P. bracteatum chr3
P. rhoeas chr3
P. bracteatum chr7
P. rhoeas chr7
C
Neo-array
P. bracteatum chr1
P. rhoeas chr1
Satellites
PCEN238
αPCEN168
PCEN338
αPCEN169
βPCEN169
βPCEN168
D
P. rhoeas chr7
P. bracteatum chr7
chr7: synteny loss region
P. setigerum chr7
P. rhoeas chr3
P. bracteatum chr3
chr3: synteny loss region
P. setigerum chr15
Gene density
LINE density
LTR density
The mean value
P. bracteatum
P. rhoeas
E
Total size comparision of LINE
Total size (Mb)
0
4
8
12
2.81
1.51
10.3
10.41
3.6
1.11
9.18
10.57
chr3: synteny loss region
chr3: other region
chr7: synteny loss region
chr7: other region
Total size comparision of LTR
Total size (Mb)
0
50
100
150
54.41
33.69
141.52
142.35
58.6
25.49
133.2
141.87
chr3: synteny loss region
chr3: other region
chr7: synteny loss region
chr7: other region

Figure S20. Syntenic conservation analysis of different centromere array patterns, related to Figure 3. (**A**) Syntenic dotplots of four chromosome pairs with retained array loci. (**B**) Syntenic dotplots of two chromosome pairs with seeded-array. (**C**) Syntenic dotplot of chromosome pair with neo-array. We labeled centromere satellites on all chromosomes. (**D**) The transposable elements (TE) distribution of chromosome pairs with seed-array. We labeled the seed array regions and syntenic loss regions as green dash boxes and gray dash boxes, respectively. (**E**) LINE and LTR TE size comparison between *P. bracteatum* and *P. rhoeas* in syntenic loss regions and other regions.

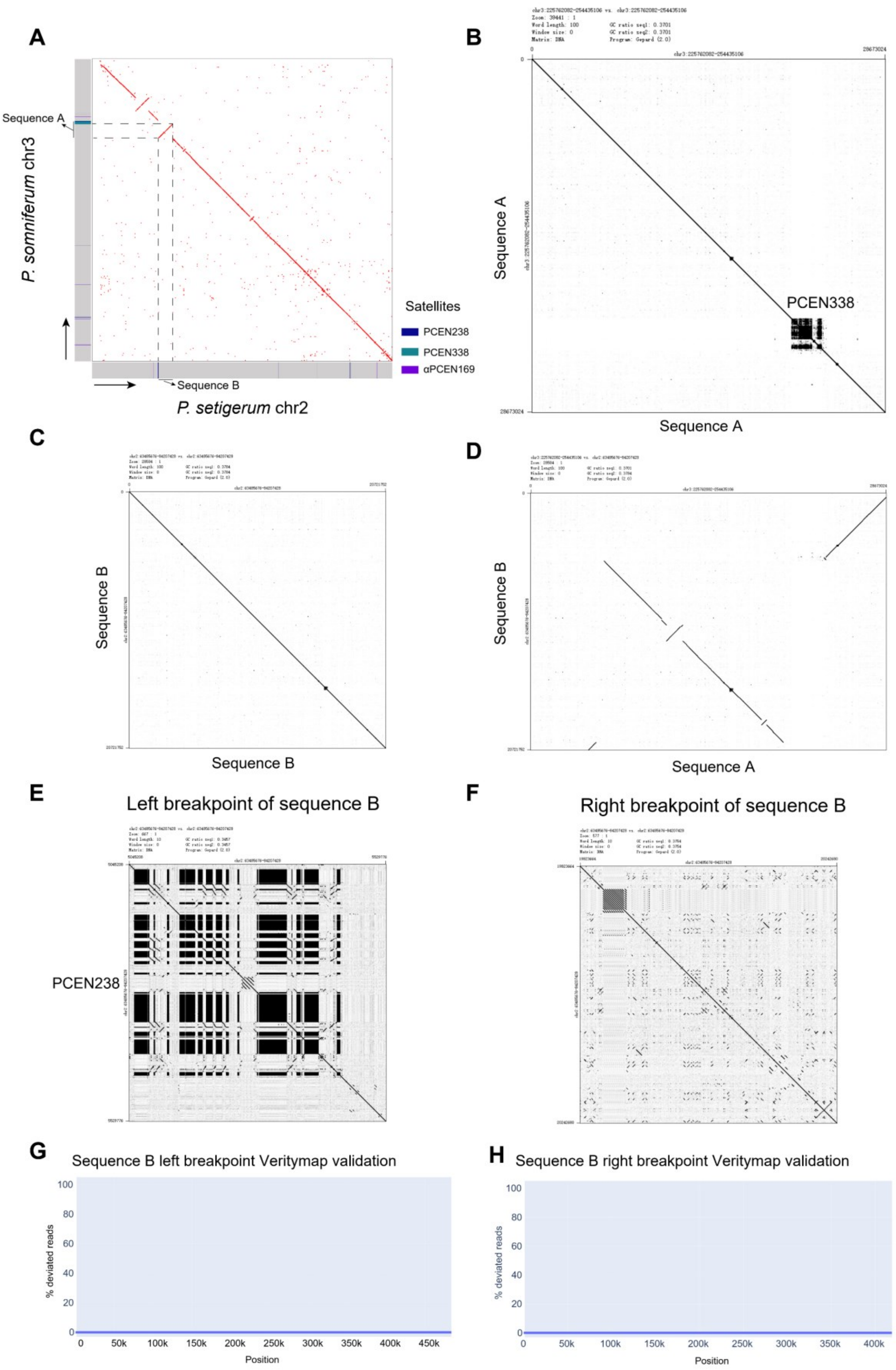
A
Sequence A
P. somniferum chr3
Sequence B
P. setigerum chr2
Satellites
PCEN238
PCEN338
αPCEN169
B
chr3:225762082-254435106 vs. chr3:225762082-254435106
Zoom: 39441 : 1
Word Length: 100
Window size: 0
Matrix: DNA
GC ratio seg1: 0.3701
GC ratio seg2: 0.3701
Program: Gepard (2.0)
Sequence A
PCEN338
Sequence A
C
Sequence B
Sequence B
D
Sequence B
Sequence A
E
Left breakpoint of sequence B
PCEN238
F
Right breakpoint of sequence B
G
Sequence B left breakpoint Veritymap validation
% deviated reads
Position
H
Sequence B right breakpoint Veritymap validation
% deviated reads
Position

Figure S21. A deletion-inversion event contributed to the loss of the PCEN338 array from *P. setigerum* chr2, related to Figure 3. (A) The syntenic dotplot between *P. somniferum* chr3 and *P. setigerum* chr2. We labeled the centromere satellites on both chromosomes. We labeled associated sequence as A and B. (**B-D**) The genomic sequence dotplots of sequence A self-comparison (**B**), sequence B self-comparison (**C**), and comparison between sequence A and B (**D**). (**E-F**) The genomic sequence dotplots of left (**E**) and right (**F**) breakpoints of sequence B. (**G-H**) The assembly validation of genomic sequence of left (**G**) and right (**H**) breakpoints of sequence B by VerityMap (v2.0.0).

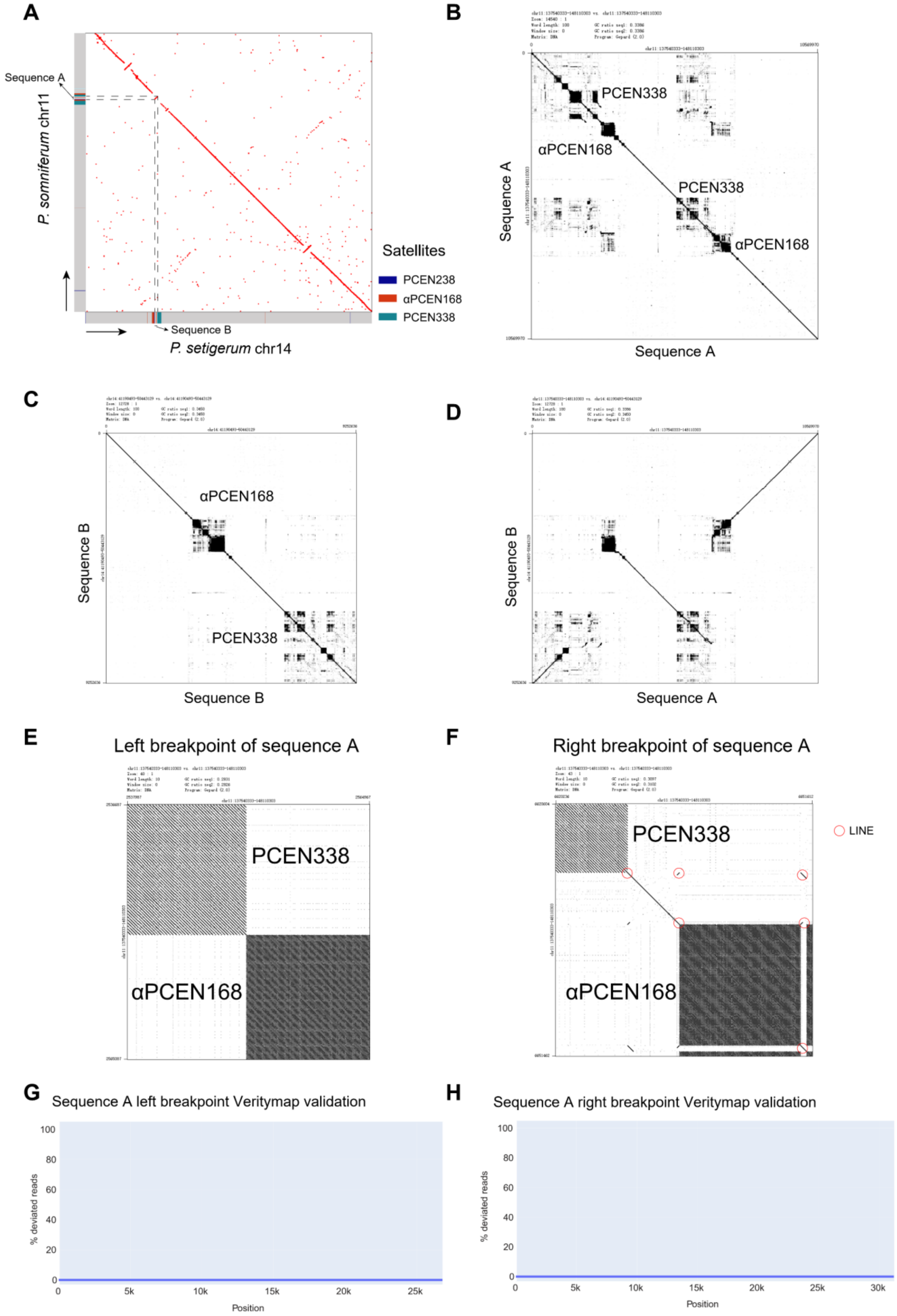
A
Sequence A
P. somniferum chr11
Sequence B
P. setigerum chr14
Satellites
PCEN238
αPCEN168
PCEN338
B
PCEN338
αPCEN168
PCEN338
αPCEN168
Sequence A
Sequence A
C
αPCEN168
PCEN338
Sequence B
Sequence B
D
Sequence B
Sequence A
E
Left breakpoint of sequence A
PCEN338
αPCEN168
F
Right breakpoint of sequence A
PCEN338
αPCEN168
LINE
G
Sequence A left breakpoint Veritymap validation
% deviated reads
Position
H
Sequence A right breakpoint Veritymap validation
% deviated reads
Position

Figure S22. **An inversion event in *P. somniferum* chr11 rearranged the satellite array compared to its syntenic counterpart, *P. setigerum* chr14, related to Figure 3.** **(A)** The syntenic dotplot between *P. somniferum* chr11 and *P. setigerum* chr14. We labeled the centromere satellites on both chromosomes. We labeled associated sequence as A and B. (**B-D**) The genomic sequence dotplots of sequence A self-comparison (**B**), sequence B self-comparison (**C**), and comparison between sequence A and B (**D**). (**E-F**) The genomic sequence dotplots of left (**E**) and right (**F**) breakpoints of sequence B. We labeled the LINE transposable element. (**G-H**) The assembly validation of genomic sequence of left (**G**) and right (**H**) breakpoints of sequence A by VerityMap (v2.0.0).

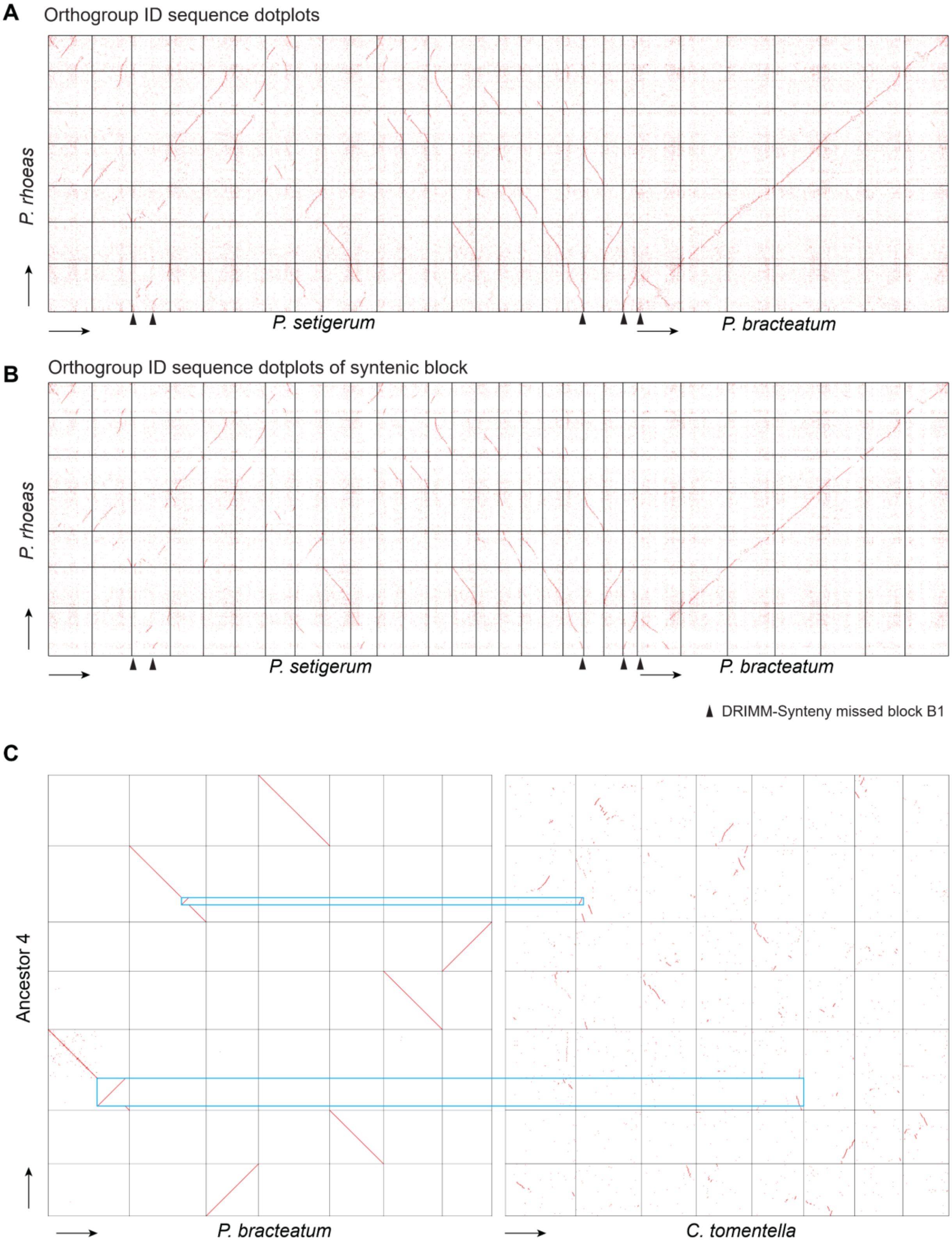

A
Orthogroup ID sequence dotplots
P. rhoeas
P. setigerum
P. bracteatum
B
Orthogroup ID sequence dotplots of syntenic block
P. rhoeas
P. setigerum
P. bracteatum
DRIMM-Synteny missed block B1
C
Ancestor 4
P. bracteatum
C. tomentella

Figure S23. Syntenic blocks used to reconstruct ancestor genomes, related to Figure 4. (A) Initial Orthogroup ID sequence dotplots between *P. rhoeas* and *P. setigerum*, and between *P. rhoeas* and *P. bracteatum*. We omitted the dotplots between *P. rhoeas* and *P. somniferum* in this figure since the genome structure of *P. somniferum* is reflected in *P. setigerum*. (**B**) The dotplots of orthogroup ID sequence from the second round syntenic blocks. We labeled the DRIMM-Synteny missed block (B1) as black triangle. (**C**) The final syntenic block dotplot of ancestor 4 with *P. bracteatum* and *C. tomentella*. Two inversions were labeled by blue boxes.

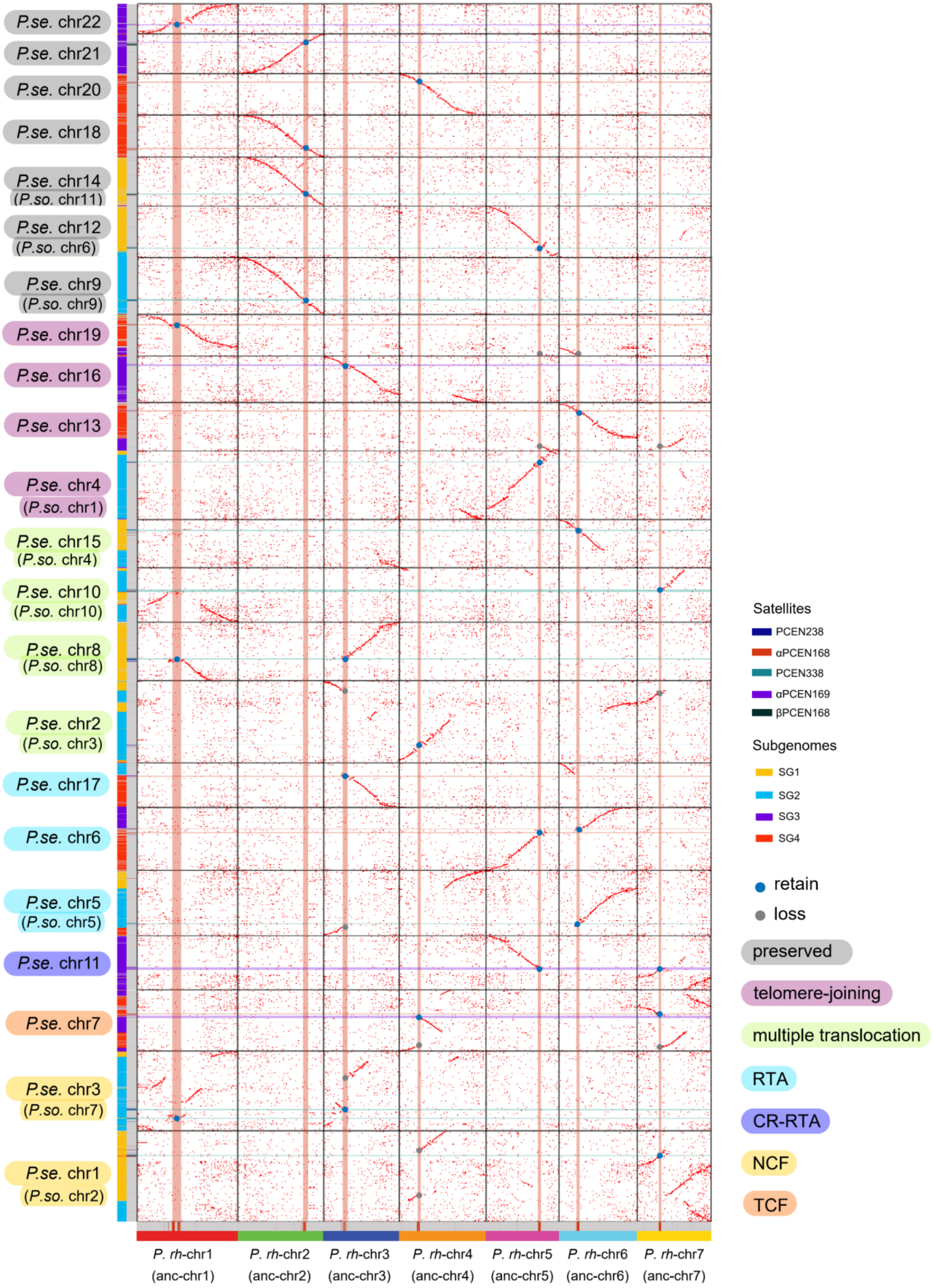

P.se. chr22
P.se. chr21
P.se. chr20
P.se. chr18
P.se. chr14 (P.so. chr11)
P.se. chr12 (P.so. chr6)
P.se. chr9 (P.so. chr9)
P.se. chr19
P.se. chr16
P.se. chr13
P.se. chr4 (P.so. chr1)
P.se. chr15 (P.so. chr4)
P.se. chr10 (P.so. chr10)
P.se. chr8 (P.so. chr8)
P.se. chr2 (P.so. chr3)
P.se. chr17
P.se. chr6
P.se. chr5 (P.so. chr5)
P.se. chr11
P.se. chr7
P.se. chr3 (P.so. chr7)
P.se. chr1 (P.so. chr2)
P. rh-chr1 (anc-chr1)
P. rh-chr2 (anc-chr2)
P. rh-chr3 (anc-chr3)
P. rh-chr4 (anc-chr4)
P. rh-chr5 (anc-chr5)
P. rh-chr6 (anc-chr6)
P. rh-chr7 (anc-chr7)
Satellites
PCEN238
αPCEN168
PCEN338
αPCEN169
βPCEN168
Subgenomes
SG1
SG2
SG3
SG4
retain
loss
preserved
telomere-joining
multiple translocation
RTA
CR-RTA
NCF
TCF

Figure S24. The syntenic dotplot between *P. setigerum* and ancestor 4, related to Figure 5. Centromere satellite locations, subgenome-phasing and syntenic dotplot between *P. setigerum* and the MRCA of four subgenomes (ancestor 4 genome is represented by *P. rhoeas* since we did not detect any rearrangement between them). The centromere satellites retain or loss was labeled based on the syntenic relations. We labeled and reordered *P. setigerum* chromosomes by their rearrangement types.

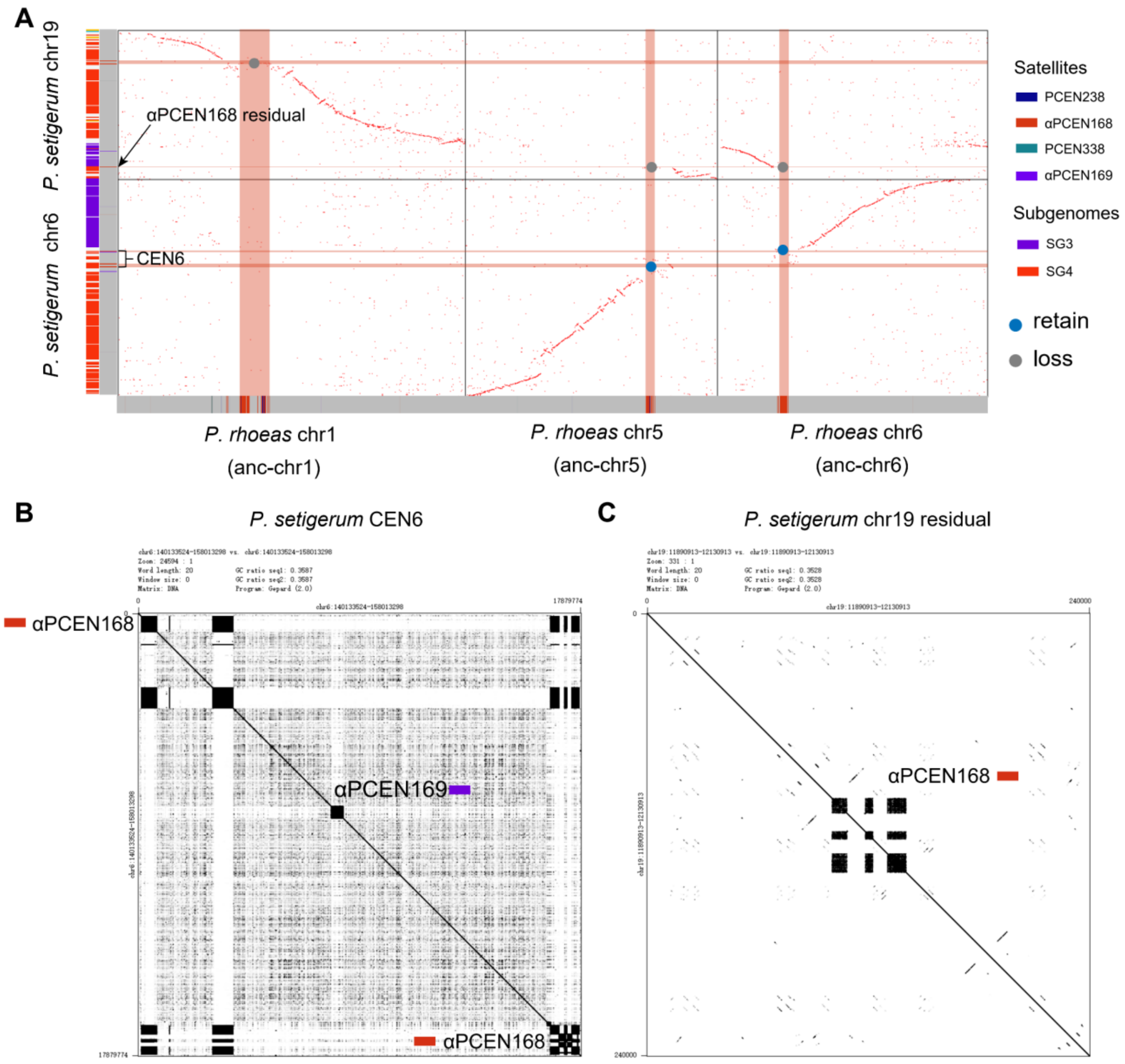

Figure S25. The centromere satellite feature of *P. setigerum* chr6 and chr19, related to Figure 5. (A) The syntenic dotplot between *P. setigerum* chr6 (Pse-chr6), Pse-chr19 and ancestor 4 chr1, chr5, and chr6 (ancestor 4 genome is represented by *P. rhoeas* since we did not detect any rearrangement between them). We labeled subgenome phasing results for Pse-chr6 and Pse-chr19. We labeled centromere satellites on all chromosomes. The centromere satellites retain or loss was labeled based on the syntenic relations. (**B-C**) The genomic sequence dotplot of CEN6 (**B**) and αPCEN168 remnant region (**C**) (regions labeled in panel **A**).

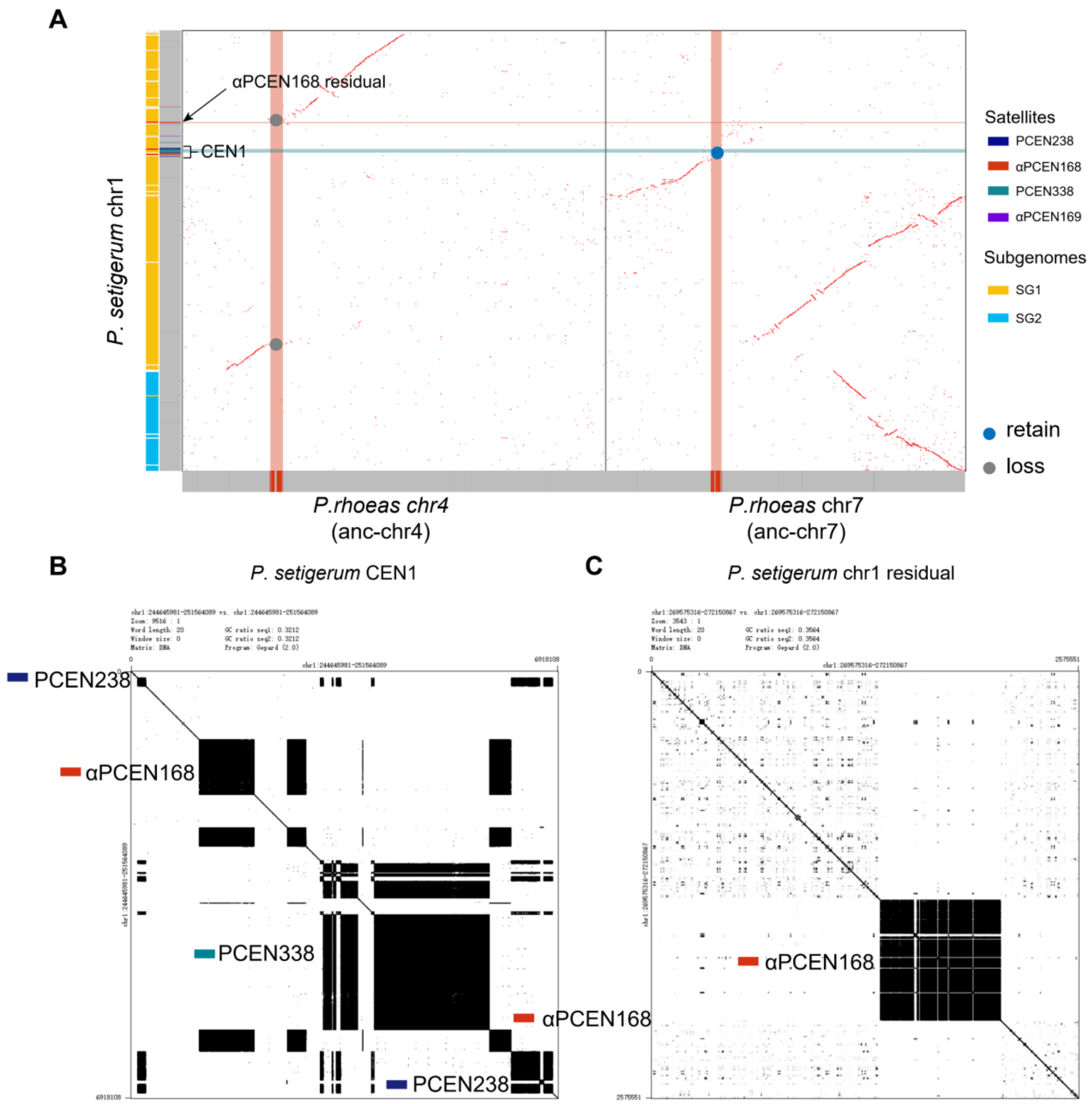

Figure S26. The centromere satellite feature of *P. setigerum* chr1, related to Figure 5. (A) The syntenic dotplot between *P. setigerum* chr1 (Pse-chr1) and ancestor 4 chr4 and chr7 (ancestor 4 genome is represented by *P. rhoeas* since we did not detect any rearrangement between them). We labeled subgenome phasing results for Pse-chr1. We labeled centromere satellites on all chromosomes. The centromere satellites retain or loss was labeled based on the syntenic relations. (**B-C**) The genomic sequence dotplot of CEN1 (**B**) and Prh168S1 remnant region (**C**) (regions labeled in panel **A**).

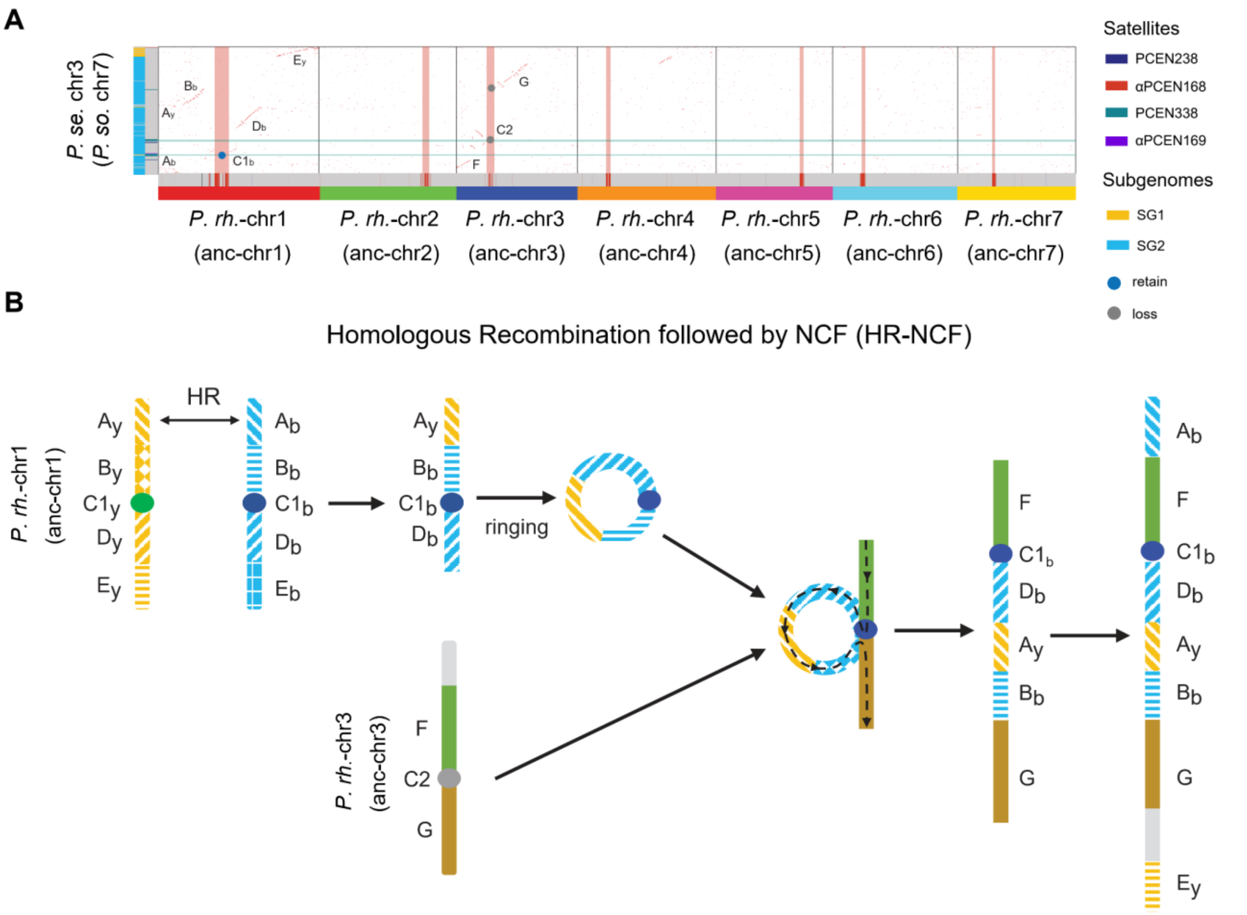

Figure S27. The centromere mediated chromosome rearrangements type of HR-NCF, related to Figure 5. (**A**) Centromere satellite locations, subgenome-phasing and syntenic dotplot between *P. setigerum* chr3 and the MRCA of four subgenomes (ancestor 4 genome is represented by *P. rhoeas* since we did not detect any rearrangement between them). The centromere satellites retain or loss was labeled based on the syntenic relations. The whole dotplot is in Figure S24. (**B**) The formation of *P. setigerum* chr3 following homologous recombination followed nested chromosome fusion (HR-NCF). *P. se.*: *P. setigerum*, *P. so.*: *P. somniferum, P. rh.*: *P. rhoeas*.

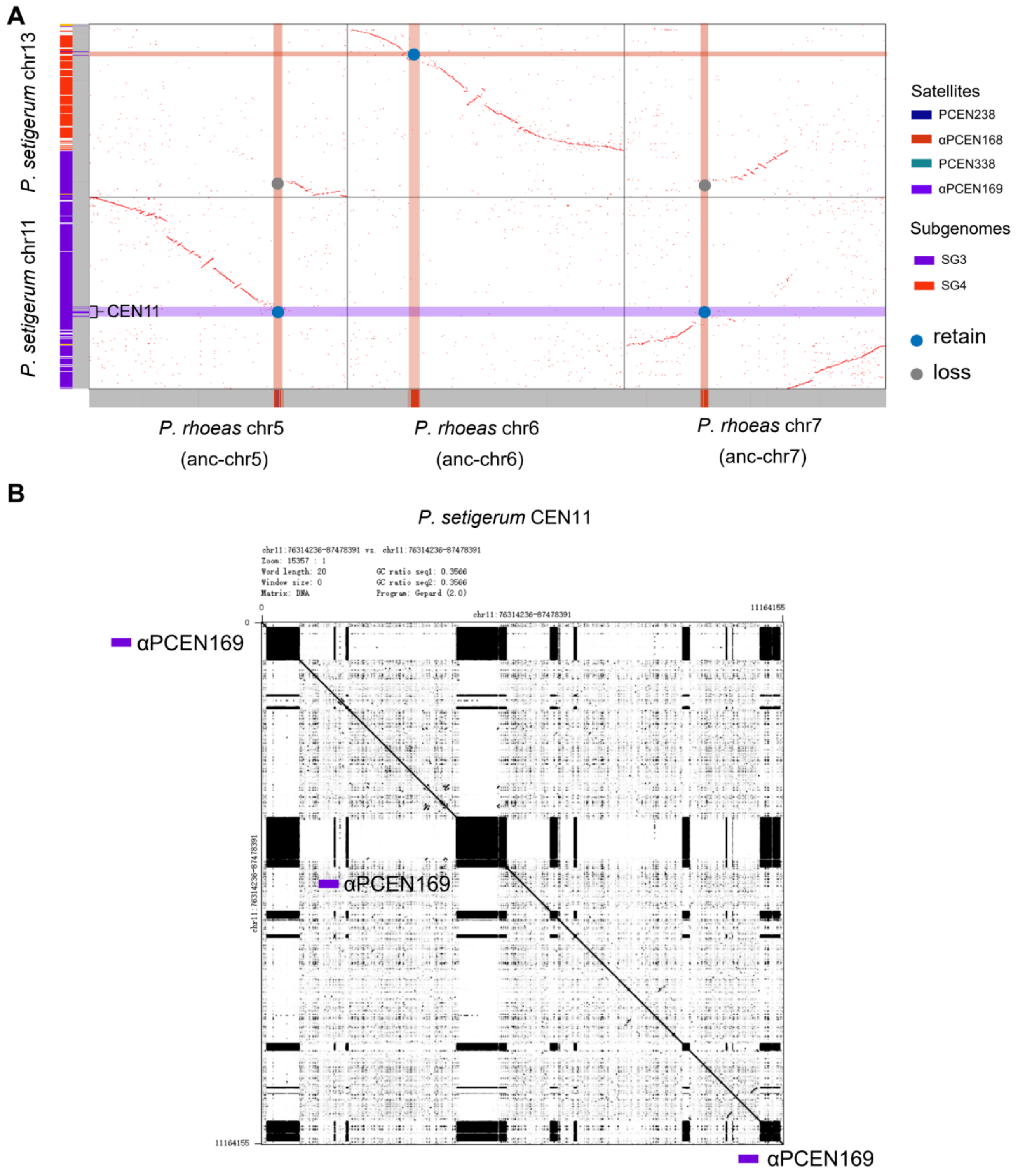

Figure S28. The centromere satellite feature of *P. setigerum* chr11 and chr13, related to Figure 5. (A) The syntenic dotplot between *P. setigerum* chr11 (Pse-chr11), Pse-chr13 and ancestor 4 chr1, chr5, and chr6 (ancestor 4 genome is represented by *P. rhoeas* since we did not detect any rearrangement between them). We labeled subgenome phasing results for Pse-chr11 and Pse-chr13. We labeled centromere

satellites on all chromosomes. The centromere satellites retain or loss was labeled based on the syntenic relations. **(B)** The genomic sequence dotplot of CEN11 (region labeled in panel **A**).

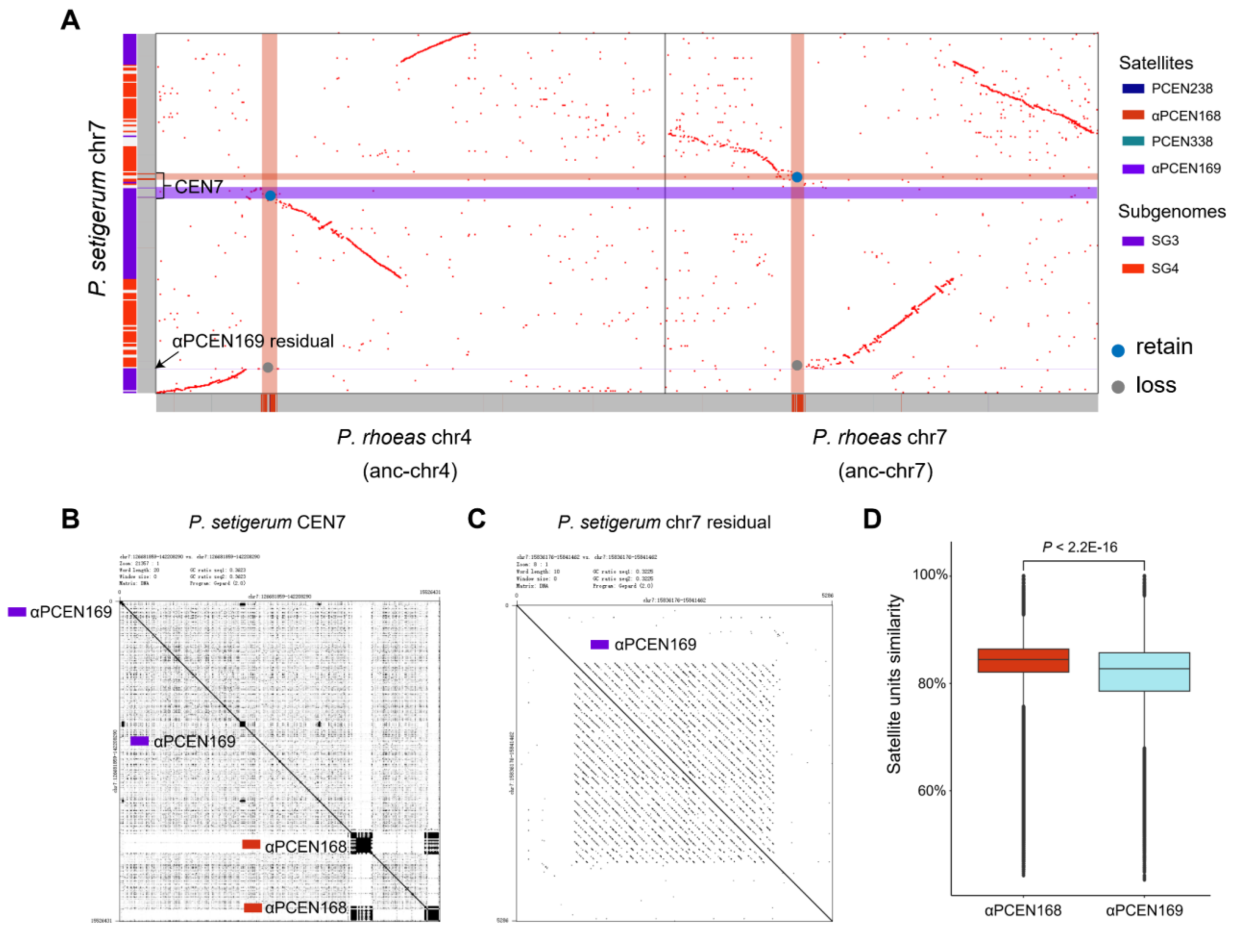

Figure S29. The centromere satellite feature of *P. setigerum* chr7, related to Figure 5. (A). The syntenic dotplot between *P. setigerum* chr7 (Pse-chr7) and ancestor 4 chr4 and chr7 (ancestor 4 genome is represented by *P. rhoeas* since we did not detect any rearrangement between them). We labeled subgenome phasing results for Pse-chr7. We labeled centromere satellites on all chromosomes. The centromere satellites retain or loss was labeled based on the syntenic relations. (**B-C**) The genomic sequence dotplot of CEN7 (**B**) and αPCEN169 remnant region (**C**) (regions labeled in panel **A**). (**D**). The sequence similarity among repeat units in αPCEN168 array was significantly higher than that of the αPCEN169 array (*p*-value < 2.2E-16, two-sided Wilcoxon rank-sum test).

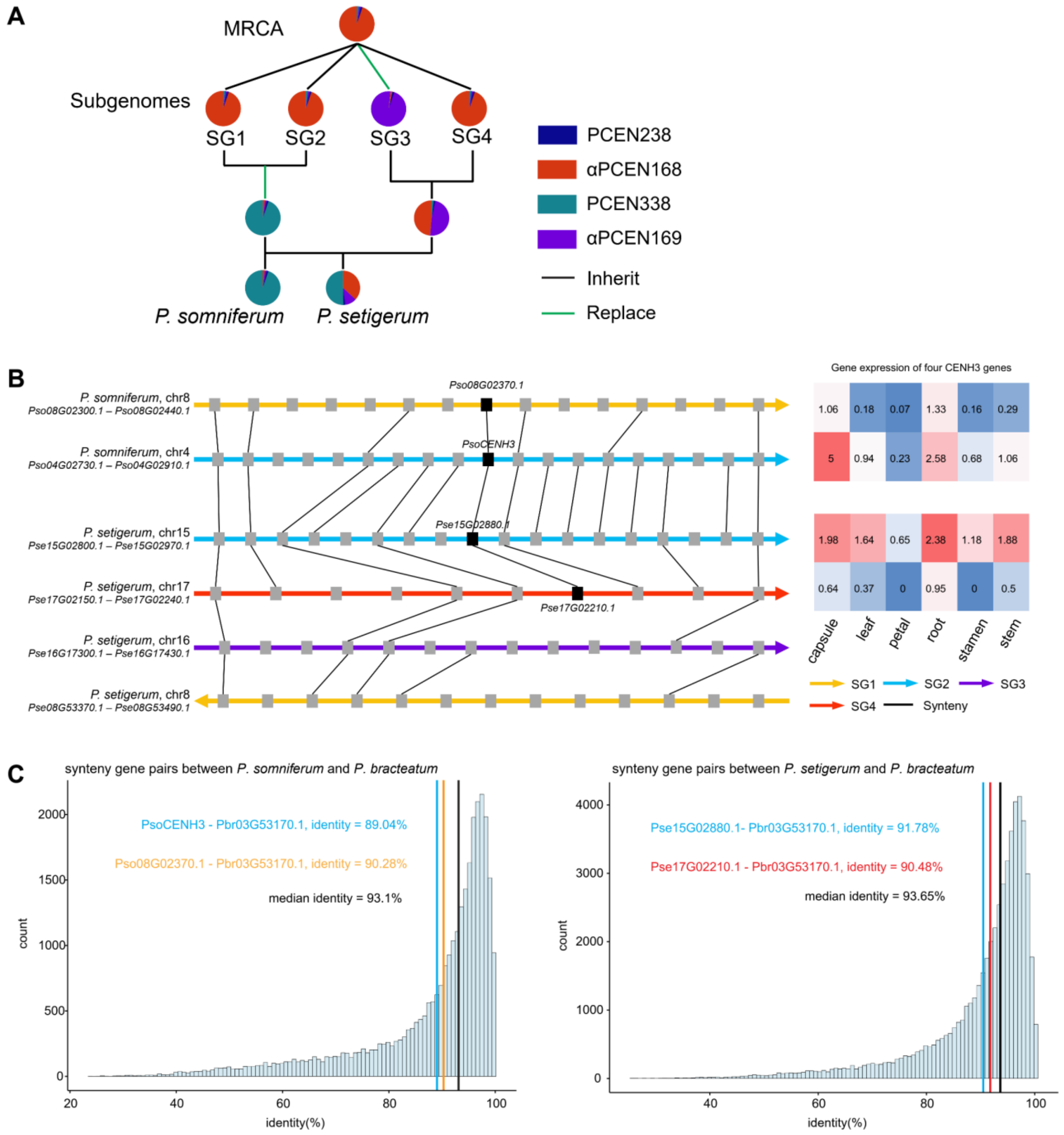

Figure S30. Centromere satellite (cenSat) competition and the coevolution of them with CENH3 in *Papaver*, related to Figure 6 (**A**) The proposed subgenome phylogenetic tree showing the centromere satellite (cenSat) competition in two alloploidydization species, *P. somniferum* and *P. setigerum*. “Replace” lines indicate that the cenSats in offspring replaced the cenSats in ancestor through satellite expansion, while “Inherit” lines indicate that the cenSats in offspring inherit from its ancestor. The order of the

hybridization between SG1 and SG2 and the order of the hybridization between SG3 and SG4 were unknown. Here, we assumed that hybridization between SG1 and SG2 happened before the hybridization between SG3 and SG4 since the almost complete competition in the dark green nodes. (**B**) The coevolution of cenSats and CENH3 in *Papaver*. **Left panel:** The syntenic relations surround *PsoCENH3* gene loci in *P. somniferum* and *P. setigerum*. Solid black lines denote the syntenic relations detected by MCScanX. Colored arrows denote different subgenomes. Black boxes represent *PsoCENH3* gene and its syntenic pair copies, and the gray boxes denote other genes. The subgenome arrow directions indicate the chromosome from 5′ to 3′. **Right panel:** The heatmap on right indicate the gene expression levels measured by TPM (transcripts per million) of four *CENH3* gene copies. Blue and red indicate low and high expression, respectively. (**C**) The amino acid sequence identity for syntenic gene pairs between *P. bracteatum* and *P. somniferum*, as well as between *P. bracteatum* and *P. setigerum*, highlighting the PsoCENH3-related gene pairs and the median value.

	Illumina paired-end short read	HiFi long-reads	ONT long-reads
P. somniferum	99.60%	99.98%	99.75%
P. setigerum	99.73%	100.00%	99.60%
P. rhoeas	97.86%	99.99%	99.63%
P. bracteatum	99.81%	100.00%	/

Table S3. Mapping rate of short and long reads to the assembled genomes, related to Table 1.

short read was aligned by bwa mem

long read was aligned by minimap2 v2.26 with --secondary=no

Mapping rate was calculated by samtools flagstat command

Chromosome	Start	End	Satellite	fold of ChIP/input
chr1	119,772,951	166,516,112	Prh168S1	8.33
chr2	241,749,882	264,735,289	Prh168S1	10.09
chr3	68,719,990	92,389,232	Prh168S1	11.69
chr4	67,924,165	78,236,448	Prh168S1	11.71
chr5	197,636,796	207,521,759	Prh168S1	8.36
chr6	65,269,046	77,660,789	Prh168S1	12.33
chr7	81,896,211	90,417,682	Prh168S1	12.27

Table S7. The inferred locations of centromere satellite arrays in *P. rhoeas*, related to Figure 2.

Papaver bracteatum			
Chromosome	Start	End	Satellite
chr1	162,089,895	176,235,833	Pbr169S4
chr2	293,846,190	304,310,528	Pbr238S1
chr3	113,315,329	114,107,637	Pbr238S1
chr4	92,707,378	112,485,024	Pbr238S1
chr5	237,250,173	257,622,058	Pbr238S1
chr6	75,197,471	86,266,469	Pbr238S1
chr7	/	/	/

Papaver somniferum				
Chromosome	Start	End	Satellite	fold of ChIP/input
chr1	216,124,564	219,416,768	Pso338S1	4.93
chr2	89,441,591	98,754,542	Pso338S1	3.20
chr3	246,747,430	249,340,990	Pso338S1	5.67
chr4	139,258,136	141,667,995	Pso338S1	4.89
chr5	59,834,744	63,358,900	Pso338S1	4.82
chr6	36,855,838	41,719,960	Pso338S1	3.30
chr7.1	221,777,281	229,638,117	Pso338S1	1.43
chr7.2	257,942,813	270,354,529	Pso338S1	2.61
chr8	140,122,150	144,753,134	Pso338S1	5.00
chr9	71,324,759	75,783,810	Pso338S1	4.84
chr10	86,150,801	96,524,632	Pso338S1	3.70
chr11	137,540,333	145,042,997	Pso338S1	3.57

Papaver setigerum			
Chromosome	Start	End	Satellite
chr1	247,753,007.0	255,847,687.0	Pse338S1
chr2	/	/	/
chr3.1	44,401,230.0	48,247,806.0	Pse338S1
chr3.2	75,706,411.0	83,581,366.0	Pse338S1
chr4	216,848,209.0	218,941,979.0	Pse338S1
chr5	48,157,359.0	50,011,543.0	Pse338S1
chr8	79,698,587.0	82,679,796.0	Pse338S1
chr9	53,383,727.0	59,020,961.0	Pse338S1
chr10	116,090,104.0	126,135,390.0	Pse338S1
chr12	38,544,295.0	43,307,296.0	Pse338S1
chr14	44,213,382.0	50,443,246.0	Pse338S1
chr15	127,900,878.0	138,938,581.0	Pse338S1
chr17	115,409,117.0	119,307,249.0	Pse168S7
chr18	28,858,146.0	35,905,843.0	Pse168S7
chr20	124,582,584.0	129,309,526.0	Pse168S7
chr6	140,233,524.0	157,913,298.0	Pse168S7
chr7	110,750,293.0	142,108,290.0	Pse168S7
chr13	150,765,457.0	156,943,220.0	Pse168S7
chr19	124,464,664.0	128,701,651.0	Pse168S7
chr11	76,414,236.0	87,378,391.0	Pse169S11
chr16	138,786,129.0	166,038,861.0	Pse169S11
chr22	32,811,185.0	38,113,270.0	Pse169S11
chr21	107,440,435.0	115,586,578.0	Pse168S13

Table S10. The inferred locations of centromere satellite arrays in *P. bracteatum*, *P. somniferum*, and *P. setigerum*, related to Figure 2.
